# Increased precipitation decelerates temporal succession of grassland soil microbial communities

**DOI:** 10.1093/ismejo/wrag176

**Published:** 2026-07-12

**Authors:** Sihang Deng, Weiling Shi, Renmao Tian, Xue Guo, Daliang Ning, Xishu Zhou, Mengting Yuan, Jiajie Feng, Aifen Zhou, Ying Fu, Gang Xu, Dashuai Mu, Peng Shi, Xiangyu Fan, Yue Teng, Xin Zhao, Zhongfang Li, Jiantao Liu, Xiaonan Liu, Liyou Wu, Zhili He, Xueduan Liu, Yiqi Luo, James M Tiedje, Yunfeng Yang, Jizhong Zhou

**Affiliations:** State Key Laboratory of Regional Environment and Sustainability, School of Environment, Tsinghua University, Beijing 100084, China; Institute for Environmental Genomics, School of Biological Sciences, University of Oklahoma, Norman, OK 73019, United States; Institute for Environmental Genomics, School of Biological Sciences, University of Oklahoma, Norman, OK 73019, United States; Institute for Environmental Genomics, School of Biological Sciences, University of Oklahoma, Norman, OK 73019, United States; State Key Laboratory of Regional and Urban Ecology, Research Center for Eco-Environmental Sciences, Chinese Academy of Sciences, Beijing 100085, China; Institute for Environmental Genomics, School of Biological Sciences, University of Oklahoma, Norman, OK 73019, United States; Institute for Environmental Genomics, School of Biological Sciences, University of Oklahoma, Norman, OK 73019, United States; School of Minerals Processing and Bioengineering, Central South University, Changsha 410083, China; Institute for Environmental Genomics, School of Biological Sciences, University of Oklahoma, Norman, OK 73019, United States; Pacific Biosciences Research Center, University of Hawai'i at Manoa, Honolulu, HI 96822, United States; Institute for Environmental Genomics, School of Biological Sciences, University of Oklahoma, Norman, OK 73019, United States; Institute for Environmental Genomics, School of Biological Sciences, University of Oklahoma, Norman, OK 73019, United States; Institute for Environmental Genomics, School of Biological Sciences, University of Oklahoma, Norman, OK 73019, United States; Institute for Environmental Genomics, School of Biological Sciences, University of Oklahoma, Norman, OK 73019, United States; School of Life Science and Engineering, Southwest University of Science and Technology, Mianyang, Sichuan 621010, China; Institute for Environmental Genomics, School of Biological Sciences, University of Oklahoma, Norman, OK 73019, United States; State Key Laboratory of Microbial Technology, Institute of Microbial Technology, Shandong University, Qingdao, Shandong 266237, China; Institute for Environmental Genomics, School of Biological Sciences, University of Oklahoma, Norman, OK 73019, United States; State Key Laboratory of Crop Stress Biology in Arid Areas, Shaanxi Key Laboratory of Agricultural and Environmental Microbiology, College of Life Sciences, Northwest A&F University, Yangling, Shaanxi 712100, China; Institute for Environmental Genomics, School of Biological Sciences, University of Oklahoma, Norman, OK 73019, United States; School of Biological Science and Technology, University of Jinan, Jinan, Shandong 250022, China; Institute for Environmental Genomics, School of Biological Sciences, University of Oklahoma, Norman, OK 73019, United States; College of Environmental and Civil Engineering, Jiangsu Key Laboratory of Anaerobic Biotechnology, Jiangnan University, Wuxi, Jiangsu 214122, China; Institute for Environmental Genomics, School of Biological Sciences, University of Oklahoma, Norman, OK 73019, United States; School of Resources and Civil Engineering, Northeastern University, Shenyang, Liaoning 110819, China; Institute for Environmental Genomics, School of Biological Sciences, University of Oklahoma, Norman, OK 73019, United States; College of Food and Bioengineering, Hezhou University, Hezhou, Guangxi 542899, China; Institute for Environmental Genomics, School of Biological Sciences, University of Oklahoma, Norman, OK 73019, United States; Key Laboratory of Natural Microbial Medicine Research of Jiangxi Province, College of Life Sciences, Jiangxi Science and Technology Normal University, Nanchang 330038, China; Institute for Environmental Genomics, School of Biological Sciences, University of Oklahoma, Norman, OK 73019, United States; Institute for Environmental Genomics, School of Biological Sciences, University of Oklahoma, Norman, OK 73019, United States; Institute for Environmental Genomics, School of Biological Sciences, University of Oklahoma, Norman, OK 73019, United States; Environmental Microbiomics Research Center, School of Environmental Science and Engineering, Southern Marine Science and Engineering Guangdong Laboratory (Zhuhai), State Key Laboratory of Biocontrol, Sun Yat-sen University, Guangzhou 510275, China; School of Minerals Processing and Bioengineering, Central South University, Changsha 410083, China; School of Integrative Plant Science, Cornell University, Ithaca, NY 14853, United States; Center for Microbial Ecology, Michigan State University, East Lansing, MI 48824, United States; Institute of Environment and Ecology, Tsinghua Shenzhen Internationl Graduate School, Tsinghua University, Shenzhen 518055, China; Institute for Environmental Genomics, School of Biological Sciences, University of Oklahoma, Norman, OK 73019, United States; School of Civil Engineering and Environmental Sciences, University of Oklahoma, Norman, OK 73019, United States; School of Computer Science, University of Oklahoma, Norman, OK 73019, United States; Earth and Environmental Sciences, Lawrence Berkeley National Laboratory, Berkeley, CA 94720, United States

**Keywords:** increased precipitation, grassland, soil microorganisms, temporal succession, functional genes, heterotrophic respiration

## Abstract

Global precipitation regimes have been shifted in recent decades, imposing significant consequences in water-limited grassland ecosystems. However, the effects of increased precipitation on the succession of soil microbial communities remain unclear, mainly due to the scarcity of long-term experiments with time-series data. Here, we examined temporal succession of grassland soil microbial communities in a long-term increased precipitation experiment. Both soil microbial taxonomic and functional structures were significantly altered by increased precipitation. Increased precipitation significantly decelerated the succession rates of soil microbial functional structure (i.e. time–decay relationships). Consistent with the increased microbial decomposition and heterotrophic respiration, the abundances of soil microbial carbon decomposition genes were markedly enhanced by increased precipitation. Furthermore, increased precipitation stimulated genes involved in nutrient cycling processes, potentially promoting plant growth. Collectively, the contributions of stochastic processes in shaping microbial communities were increased under increased precipitation, suggesting that microbial successional trajectories may shift toward multiple alternative states characterized by greater stochasticity under future altered precipitation regimes.

## Graphical Abstract

**Graphical Abstract:**
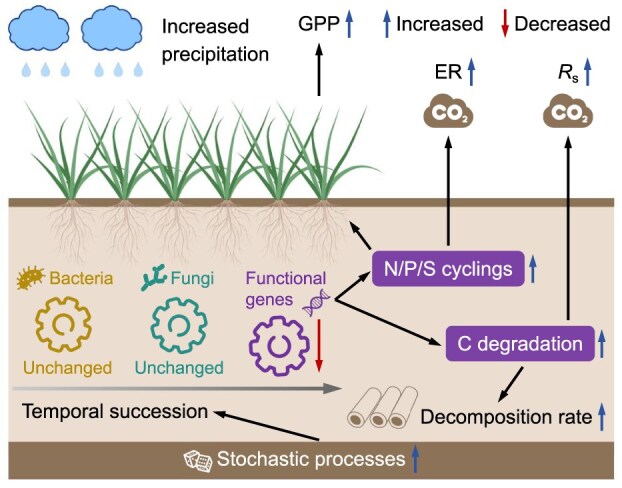

## Introduction

Climate change, such as warming and altered precipitation regimes, has been evidenced globally and can substantially impact the terrestrial ecosystem processes [1, 2]. Global warming has intensified the shifts in global precipitation patterns [3]. General circulation models predict that precipitation increases or decreases, depending on geographic locations, with increased interannual variability of precipitation and a higher frequency of extreme wet and dry years in the future [3, 4]. Such alterations in precipitation patterns may have particularly significant consequences in water-limited grassland ecosystems, which cover 20%–40% of the terrestrial land surface and provide important ecosystem services [5, 6].

In the last two decades, various precipitation manipulation experiments have been conducted to determine how grassland ecosystems respond to precipitation alternations. However, most of them are largely focused on the responses of plant communities to altered precipitation, especially the aboveground net primary productivity (ANPP) [7, 8]. Despite microorganisms being among the most abundant and diverse life forms on Earth and key mediators of biogeochemical cycles (C, N, P, S), much less is known about the responses of belowground soil microbial communities to precipitation changes in grassland ecosystems. This knowledge gap is further underscored by our recent findings that microbial communities modulate the effects of warming on topsoil organic carbon under varying precipitation conditions, highlighting the urgent need to study them [9]. However, due to extremely high diversity and complexity of soil microbial communities and technical challenges in examining microbial community composition and function, previous studies were primarily focused on investigating the responses of microbial aggregate properties (e.g. microbial biomass, enzyme activities, or soil respiration) to altered precipitation [10–12]. A few recent studies examined shifts in soil microbial community structure in response to precipitation alterations, but their findings remain inconclusive [13–15], largely due to the reliance on single-time-point sampling. For instance, soil microbial community structure showed no significant response to either increased or decreased precipitation over a 1-year period in semiarid woodlands [16]. A seasonal precipitation manipulation experiment in a northern California grassland indicated that soil bacterial and archaeal communities remained statistically indistinguishable over 5 years of increased precipitation treatment [17]. In contrast, long-term increases in seasonal precipitation have been shown to significantly alter both bacterial and fungal community structures in a cold desert sagebrush steppe [18]. Since soil microbial communities are very sensitive to environmental changes and highly dynamic along time [19–21], it is crucial to perform time-series analyses for understanding long-term dynamics of microbial communities in response to precipitation alternations under highly variable environmental conditions.

In this study, we examined the succession of taxonomic and functional diversity of soil microbial communities in a long-term (>7 years) climate change experiment by focusing on increased precipitation (+100%) in a native, tallgrass prairie ecosystem of the US Great Plains in Central Oklahoma (34° 59′ N, 97° 31′ W) [20, 22]. The experimental site was established in 2009, and a rainfall-collection-redistribution device was used to increase precipitation by 100% [22]. Soil samples were archived in the increased precipitation and control plots every year from 2009 to 2016, and analyzed by 16S rRNA gene-based amplicon sequencing for prokaryotes, internal transcribed spacer (ITS)-based amplicon sequencing for fungi, and functional gene arrays (GeoChip 5.0M) for microbial community functional gene structure. Given that our ecosystem is located in a precipitation-limited region, here, we aim to test the following hypotheses: (i) Increased precipitation-induced environmental changes would alter the temporal succession of soil microbial taxonomic and/or functional communities; (ii) Increased precipitation could activate soil microbial communities, leading to increased soil C decomposition and nutrient (e.g. N, P, and S) cycling processes; (iii) The relative importance of deterministic processes would be reduced in shaping temporal succession of soil microbial communities, due to the alleviation of environmental stresses by increased precipitation.

## Materials and methods

### Site description and soil sampling

This study was conducted at the Kessler Atmospheric and Ecological Field Station, which is an old-field tall grass prairie located at the US Great Plain in McClain County, Oklahoma, USA (34^o^59′N, 97^o^31′W) [22]. Abandoned from cropping 40 years ago with light grazing until 2008, this field site is dominated by C_3_ forbs (e.g. *Ambrosia trifda, Solanum carolinense,* and *Euphorbia dentata*), C_3_ grasses (*Bromus spp*), and C_4_ grasses (*Tridens flavus* and *Sorghum halapense*). The mean annual temperature is 16.3°C and the mean annual precipitation is 914 mm (Oklahoma Climatological Survey from 1948 to 1999, Norman, OK, USA) [20]. The soil type of this field is Port–Pulaski–Keokuk complex, which is loam with 51% sand, 35% silt, and 14% clay. The concentrations of soil organic matter and total nitrogen (TN) are 1.9% and 0.1%, respectively, and the soil bulk density is 1.2 g/cm^3^. The soil has a high available water holding capacity (37%), neutral pH, and a deep (ca. 70 cm), moderately penetrable root zone [22].

The field site experiment was established in July of 2009, with warming (+3°C), increased precipitation (+100%), and decreased precipitation (−50%) as primary factors. The experiment design has been described in detail previously [9, 22]. Briefly, the site has four experimental blocks as four replicates, each containing six 2.5 m × 3.5 m plots (Supplementary Fig. S1). The southern halves of the plots were clipped annually, and the northern halves were unclipped. Treatments including control, warming, increased precipitation, decreased precipitation, warming with increased precipitation, and warming with decreased precipitation were randomly assigned in the six plots of each block. This study focused on the effects of increased precipitation under ambient temperature without the influence of clipping. Rainfall-collection-redistribution devices, which are angled catchments with the same size and shape as the plot, were installed to collect and redirect precipitation into plots with increased precipitation.

From 2009 to 2016, eight surface (0–15 cm) soil samples were collected annually in September or October from the unclipped halves of the plots with control (four plots) and increased precipitation (four plots) treatment. Each sample was mixed from three soil cores (2.5 cm diameter × 15 cm depth) using a soil sampler tube. Soil samples were immediately transported to the laboratory and stored at −80°C before analyses. In this study, a total of 64 annual soil samples (8 years × 8 samples per year) taken under control and increased precipitation treatment without clipping effects were further analyzed.

### Field measurements and soil physicochemical analyses

Precipitation data were obtained from an Oklahoma Mesonet Station (Washington Station) located ~200 m away from the field site. Constantan-copper thermocouples wired to a Campbell Scientific CR10x data logger (Campbell Science Inst., Logan, UT, USA) were used to measure and record soil temperature every 15 min at a depth of 7.5 cm in the center of each subplot. Volumetric soil water content (%V) from the soil surface to a 15 cm depth was measured once or twice a month using manual Time Domain Reflectometry equipment (Soil Moisture Equipment Crop., Santa Barbara, CA, USA). Three soil water content measurements were performed in each subplot each time, and the average values were used.

All soil samples were analyzed by the Soil, Water, and Forage Analytical Laboratory at Oklahoma State University (Stillwater, OK, USA). The soil total organic C (TOC) and TN were determined using a dry combustion C and N analyzer (LECO, St. Joseph, MI, USA). Soil nitrate (NO_3_^−^) and soil ammonia (NH_4_^+^) were analyzed using a Lachat 8000 flow-injection analyzer (Lachat, Milwaukee, WI, USA). Soil pH was measured at a water-to-soil mass ratio of 2.5:1 using a pH meter with a calibrated combined glass electrode.

### Aboveground plant communities

Above-ground plant biomass, divided into C_3_ and C_4_ species, was estimated annually at peak biomass (usually September) by a pin-touch method [22]. All of the species within each plot were identified to estimate species richness. The estimated above-ground plant biomass was considered to be ANPP in this study [20].

### Ecosystem carbon fluxes and soil respiration

Ecosystem carbon (C) fluxes were measured once or twice a month between 10:00 and 15:00 (local time), as described previously [22]. Ecosystem respiration (ER) and net ecosystem exchange (NEE) were measured using an LI-6400 portable photosynthesis system (LI-COR Inc., Lincoln, NE, USA) attached to a transparent chamber (0.5 m × 0.5 m × 0.7 m), which covered all the vegetation within the aluminum frames. Gross primary productivity (GPP) was estimated as the difference between NEE and ER. Soil total respiration (*R*_t_) and heterotrophic respiration (*R*_h_) were measured using a LI-8100A soil flux system attached to a soil CO_2_ flux chamber (LI-COR Inc., Lincoln, NE, USA). Autotrophic respiration (*R*_a_) was estimated as the difference between *R*_t_ and *R*_h_.

### Soil decomposition rate

The cellulose decomposition rate was measured by adding external cellulose substrate. In March 2016, 5 g of sterile cellulose filter paper (Whatman CAT No. 1442-090) was placed into fiberglass mesh bags, which were then placed vertically at a soil depth of 5–10 cm within each plot. The bags were collected in September 2016, rinsed, and dried at 60°C for weighing. The percentage of mass loss was calculated to represent decomposition rate.

### DNA extraction

Soil DNA was extracted from 1.5 g soil by freeze-grinding and SDS-based lysis as described previously [23], and purified with the MoBio Power Soil DNA isolation kit (MoBio Laboratories, Carlsbad, CA, USA). DNA quality was assessed based on the ratios of 260:280 nm and 260:230 nm absorbance using a NanoDrop ND-1000 Spectrophotometer (NanoDrop Technologies Inc., Wilmington, DE, USA). The DNA concentrations were quantified by PicoGreen using a FLUOstar Optima (BMG Labtech, Jena, Germany). The DNA samples were stored at −80°C until analysis.

### Amplicon sequencing

Universal primer sets, 515F (5′-GTGCCAGCMGCCGCGGTAA-3′) and 806R (5′-GGACTACHVGGGTWTCTAAT-3′) targeting the V4 hypervariable region of prokaryotic 16S rRNA genes [24], and gITS7F (5′-GTGARTCATCGARTCTTTG-3′) and ITS4R (5′-TCCTCCGCTTATTGATATGC-3′) for fungal ITS2 between 5.8S and 28S rRNA genes [25], were used in this study. Library construction was performed using a two-step PCR to avoid extra PCR bias [26]. Briefly, soil DNA was first diluted to 2.5 ng/μl with water to be used as a template in the first round PCR reaction. PCR products of the first-round PCR were purified using Agencourt Ampure XP (Beckman Coulter, Inc., Brea, CA, USA) and used as templates for the second-round PCR, which was carried out in triplicate. Subsequently, the triplicate products were combined, visualized by 1% agarose gel electrophoresis, and quantified by PicoGreen. PCR products from different samples were pooled at equal molality, purified with a QIAquick gel extraction kit (Qiagen Sciences, Germantown, MD, USA), and quantified with PicoGreen. Sample libraries for sequencing were prepared according to the MiSeq Reagent Kit Preparation Guide (Illumina) and sequenced on a MiSeq System (Illumina) using 2 × 250 pair-end sequencing kit following the manufacturer’s instructions.

### Sequence preprocessing

The raw reads of the 16S rRNA gene and ITS were collected by the MiSeq in fastq format and then submitted to our sequence analysis pipeline (http://zhoulab5.rccc.ou.edu:8080) built on the Galaxy platform [27] for further analysis. After removing spiked PhiX reads, the reads were assigned into different sample libraries based on the barcodes. Primer sequences at the end of each read were trimmed and the Btrim program with a quality control threshold >20 over a five base pair (bp) window size was used to filter the reads. Then, the forward and reverse reads of the same sequence with at least 20 bp overlap and <5% mismatches were combined using FLASH [28]. Any joined sequences with an ambiguous base or a length of <245 bp for the 16S rRNA gene or < 220 bp for the ITS were discarded. Subsequently, operational taxonomic units (OTUs) were clustered by UPARSE [29] at 97% identity and singletons were removed from the remaining sequences for both the 16S rRNA gene and the ITS. In UPARSE, the Greengenes reference data set (v13.5) [30] for 16S rRNA gene data and the UNITE ITS reference data set (v8.2) for ITS data were used as reference databases to remove chimeras. To normalize samples to the same total read abundance, 21 576 sequences for the 16S rRNA gene, and 7784 sequences for the ITS were randomly selected (resampled) for each sample. OTU taxonomic classification of the 16S rRNA gene and ITS sequences was performed using representative sequences from each OTU through the Ribosomal Database Project Classifier with 50% confidence estimates (v2.14) [31].

### GeoChip analyses

GeoChip 5.0M (180K) functional gene arrays, were used to analyze the functional structure of soil microbial communities across the 7 years. GeoChip 5.0M, manufactured by Agilent (Agilent Technologies Inc., Santa Clara, CA, USA), is capable of detecting and quantifying over 365 000 functional genes from 1447 gene families related to the biogeochemical cycling of C, N, S, and P, and other functional categories [32]. In our study, 800 ng of purified DNA of each of the 64 soil samples was labeled with the fluorescent dye Cy-3 (GE Healthcare, Anaheim, CA, USA) using a random priming method, purified using a QIAquick Purification kit (Qiagen, Mountain View, CA, USA), and then dried in a SpeedVac (Thermo Savant, Holbrook, NY, USA). Subsequently, the labeled DNA was suspended into 27.5 μl of DNase/RNase-free distilled water and then mixed well with 99.4 μl of hybridization solution. The mixture was denatured at 95°C for 3 min, spun at 6000 × g for 1 min, incubated at 37°C for 30 min, and hybridized with GeoChip arrays at 67°C for 24 h at 20 rpm in a hybridization oven. After hybridization, the slides were washed with Agilent hybridization buffer and then scanned with a NimbleGen MS200 Microarray Scanner (Roche NimbleGen, Inc., Madison, WI, USA). The images of the arrays were converted and extracted using Agilent Feature Extraction 11.5 software.

The microarray data of the 64 soil samples were processed using our microarray analysis system (http://ieg.ou.edu/microarray/). The major steps were as following: (i) Raw signal intensities (Cy3 channel) on each array were multiplied by a normalization weight I, which is the ratio of the maximum average universal standard intensity (Cy5 channel) among all the samples divided by the average universal standard intensity of each array; (ii) The signal intensities on each array were further multiplied by a normalization weight II, which is the ratio of the maximum total raw intensity (Cy3 channel) among all the samples divided by the total raw intensity of each array; (iii) Spots with SNR (signal-to-noise ratio) ≥ 2 were considered as positive. Otherwise, they were treated as negative spots with 0 value; (iv) Spots with signal intensity lower than 250 were not considered as positive and were removed in subsequent analysis; (v) The mean ratio in each sample was calculated by dividing the transformed signal intensity of each probe by the average transformed signal intensity for all detected probes in each sample. (vi) Relative changes in normalized signal intensities were calculated as the fold change in gene abundance under increased precipitation relative to the paired control within the same block, expressed as [(increased precipitation − control) / control], either in each year or across years.

### Statistical analyses

Various statistical analyses were carried out using *R* software (v.4.2.1) with the package *vegan* unless otherwise indicated. Differences in environmental variables, including soil properties (e.g. moisture, pH) and plant characteristics, relative abundances of microbial phyla/classes, and functional gene intensities between increased precipitation (IP) and control were assessed using repeated measures analysis of variance (ANOVA) in each year or across seven years. Additionally, one-tailed paired *t-*tests were employed to evaluate treatment effects on NO_3_^−^ and mass loss. Differences in soil microbial taxonomic and functional communities between the increased precipitation and control treatments were assessed using permutational multivariate analysis of variance (Adonis). For Adonis, the two-way repeated-measures ANOVA model was set as “Dissimilarity ~ IP × Year + Block”, using the *Adonis2* function in the R package *vegan*.

Temporal patterns of microbial community structures in the increased precipitation and control plots were determined by non-metric multidimensional scaling (NMDS) ordination, and analysis of the time–decay relationships (TDRs) based on Bray–Curtis and Sorenson dissimilarity metrics. The moving window approach was used to calculate TDRs of microbial communities [20]. In our 8-year record, there are seven 1-year intervals, six 2-year intervals, five 3-year intervals, down to one 7-year interval for each plot. Considering the repeated measures design, TDR analysis counted only pairwise comparisons among time points within each plot (resulting in 28 pairwise comparisons for each plot and a total of 112 pairwise comparisons for each treatment). Considering that our experimental design has repeated measures at different time points in the same plot, and different plots under the same treatment do not necessarily have the same slope and intercept, the data for each treatment was fitted to the TDR model by a linear mixed-effects model (LMM) [20]. The model was calculated using the function *lmer* in the R package *lme4* with model setting as ln (*S_s_*) ~ ln (d*T*) + [1 + ln (d*T*)]|plot, where *S_s_* is the pairwise similarity in community composition, and d*T* is the time interval. The slope (*v*) of the linear mixed-effects regression between log-transformed community similarity and log-transformed time interval was used as the TDR value, representing the temporal turnover rate of the microbial community. The coefficient of determination (*R* [2]) was calculated for each LMM, following Nakagawa and Schielzeth’s method (referred to as conditional *R* [2]) [33] to evaluate how the data can be explained by the TDR model. The significance of each LMM was calculated by a permutation test rather than a parametric test considering the dependence among the pairwise comparisons. The permutation test randomized the 8 time points (years) for 1000 times, and the *P* value was calculated by comparing the Akaike information criterion value of the observed LMM with the permuted ones. We also performed a permutation test to calculate the significance of the difference in TDR values between increased precipitation and control [20, 34]. The observed difference was compared with the TDR value difference in permuted datasets to obtain the *P* value.

The correlations between environmental factors and microbial community composition measured by 16S rRNA gene sequencing, ITS sequencing, and GeoChip hybridization, were examined using canonical correspondence analysis (CCA), Mantel tests, and partial least squares (PLS) modeling. For CCA, the OTU tables of 16S rRNA gene and ITS sequencing, and the functional profile of GeoChip were used as the response variables and the environmental factors including soil properties and plant characteristics were used as the explanatory variables. The significance of the CCA model was tested using ANOVA. Based on CCA results, variation partitioning analysis (VPA) was performed to determine the contributions of each group of variables to total variations in the soil microbial communities. Mantel tests were also performed to calculate the correlations between environmental factors, microbial taxonomic communities, and specific functional gene groups or individual genes, with *P*-values adjusted for multiple testing using the Benjamini–Hochberg procedure to control the false discovery rate. For PLS, microbial taxonomic and functional community composition was represented by the first axis of non-metric multidimensional scaling (NMDS1) based on Bray–Curtis dissimilarity metrics. To account for potential temporal autocorrelation, plot-level means were calculated by averaging microbial and environmental measurements across time points within each plot [35]. In each optimal PLS model, predictor variables were selected using a forward selection procedure guided by biological and biogeochemical relevance. The selection was based on predictive performance, taking into account explained variation (*R*^2^_Y_) and model significance (*p* for *R*^2^_Y_ and *Q*^2^_Y_ < 0.05, with significant *Q*^2^_Y_ serving to prevent overfitting). Consistent with previous studies [35], partial *R*^2^ indices were calculated to depict the proportion of variance explained by each independent variable. For reference, we also computed pairwise correlation coefficients (*r*) among the factors, with significance determined using Pearson correlation. All PLS analyses were performed using the *ropls* package in R [36]. A list of potential predictors (X variables) considered for each dependent variable (Y) in the PLS models is provided in Supplementary Table S1.

Normalized stochasticity ratio (NST) was demonstrated as an effective tool for quantifying the relative importance of stochastic processes in community assembly [37]. Our study utilized a special NST, also referred to as the modified stochasticity ratio (MST), which constrains pairwise values to a range of 0 to 1 [38]. MST was calculated using Bray–Curtis and Sorensen dissimilarity metrics under a null model algorithm based on proportional frequency with fixed richness, implemented with the *NST* package in R [37]. Each pairwise comparison between two samples generated an MST value. Only pairwise comparisons within the same treatment (increased precipitation or control) and sampling year were included in the analysis. Accordingly, for each treatment and sampling year, six MST values were derived from the four samples. The stochasticity of microbial community assembly under each treatment was represented by the 7-year mean MST. Treatment differences in MST were assessed using two-way repeated measures ANOVA. To investigate treatment-specific temporal dynamics, LMMs were applied separately to the increased precipitation and control groups, with block as a random effect, to test for correlations between sampling year and functional gene intensities. The significance of each LMM was determined using Wald-type II χ^2^ tests implemented in the *car* R package [39]. All *P* values were adjusted for multiple testing using the Benjamini–Hochberg procedure to control the false discovery rate.

## Results

### Effects of increased precipitation on soil variables and ecosystem C fluxes

**Figure 1:**
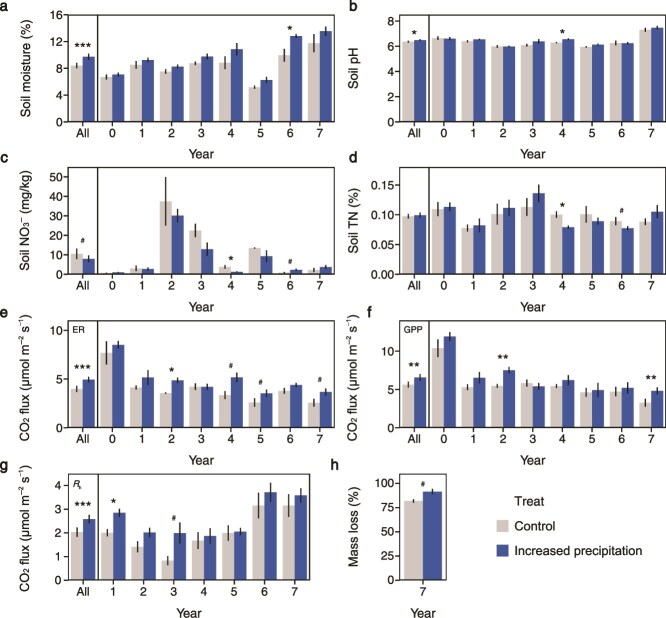
Effects of increased precipitation (IP) on soil variables and ecosystem C fluxes across seven years. (a) Soil moisture. (b) Soil pH. (c) Soil nitrate (NO_3_^−^). (d) Soil TN. Ecosystem C fluxes, which were estimated based on the C amount from CO_2_ emissions. (e) ER. (f) GPP. (g) Soil heterotrophic respiration (*R*_h_) *in situ*. (h) Decomposition rate of standard cellulose filter paper (mass loss) in the field determined in 2016. Year 0 represents the year 2009. Error bars indicate standard error. The differences between increased precipitation (blue bar) and the control (grey bar) for each year and across all years were assessed using repeated measures ANOVA. Differences in soil NO_3_^−^, TN, and mass loss were further evaluated using one-tailed paired *t*-tests. Significance levels denoted as ^***^P < .001, ^**^P < .01, ^*^P < .05, ^#^*P <* .10. No significant interaction effects between treatment and sampling year were detected for the measured soil variables or ecosystem C fluxes.

Increased precipitation significantly altered soil variables and key ecosystem processes, including ecosystem C fluxes and soil respiration (Fig. 1). Specifically, soil moisture was increased from 8.4% to 9.7% (*P <* .001) in response to increased precipitation across the seven years, with a pronounced effect in 2015 (from 9.9% to 12.8%, *P =* .031; Fig. 1a). Similarly, soil pH was increased from 6.36 to 6.49 across the 7 years (*P =* .035), particularly in 2013 (from 6.28 to 6.56, *P =* .038; Fig. 1b). No significant difference in soil temperature was observed between treatments, although a marginal decrease from 17.0°C to 16.7°C was shown in 2014 (*P =* .087; Supplementary Fig. S2a). Although soil ammonium (NH_4_^+^), and TOC remained unchanged under increased precipitation (Supplementary Fig. S2b and c), soil nitrate (NO_3_^−^) concentration was marginally decreased (one-tailed paired *t-*test, *P =* .093), particularly in 2013 (*P =* .021), which further contributes to the decrease in TN (*P =* .026; Fig. 1c and d). Over 7 years, average plant biomass and species richness exhibited no significant responses to increased precipitation, with the exception of a significant increase in total plant biomass in 2014 driven by C_4_ plants (*P <* .05; Supplementary Fig. S2d–g).

Over 7-year increased precipitation treatment enhanced ER by 23.9% (*P <* .001), and GPP by 16.6% (*P =* .004), with the most pronounced increases (*P <* .05) observed in 2011 for ER (36.8%), and in 2011 and 2016 for GPP (37.5% and 47.3%; Fig. 1e and f). Moreover, microbial decomposition of soil organic matter, measured as heterotrophic respiration (*R*_h_), was significantly (*P <* .001) increased by increased precipitation (Fig. 1g), whereas autotrophic respiration (*R*_a_) from belowground plant activity remained unchanged (Supplementary Fig. S2h). This differential response resulted in no net change in total soil respiration (*R*_t_) (Supplementary Fig. S2i). The enhanced *R*_h_ under increased precipitation suggested accelerated soil C decomposition, a hypothesis further supported by the litterbag experiment in 2016. Cellulose filter paper buried in treatment plots exhibited a higher mass loss rate compared to controls (one-tailed paired *t*-test, *P =* .058), indicating an increased decomposition rate (Fig. 1h), though this measurement was performed only in the final year. Overall, only NEE exhibited a significant interaction effect (*P =* .045) between increased precipitation and sampling year (Supplementary Fig. S2j), indicating that the effects of increased precipitation on environmental variables remained consistent, showing no progressive accumulation or interannual variability (Fig. 1, and Supplementary Fig. S2).

### Impacts of increased precipitation on the temporal succession of soil microbial communities

**Figure 2:**
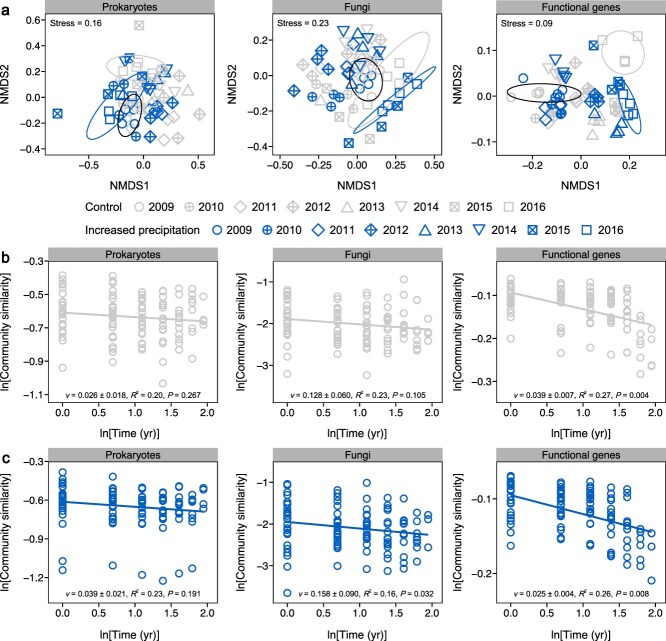
Responses of soil microbial taxonomic and functional communities to increased precipitation over time. (a) NMDS ordination based on Bray–Curtis dissimilarity illustrating temporal changes in prokaryotic (16S rRNA gene sequencing), fungal (ITS sequencing), and functional gene (GeoChip) communities under control and increased precipitation conditions. In the first year (2009), samples from both treatments clustered together (encircled in black). In subsequent years, particularly evident in 2016, communities under increased precipitation progressively separated from controls, with the most pronounced separation observed for the functional gene community (blue circle: increased precipitation; grey circle: control). The TDRs for prokaryotic, fungal, and functional gene communities under control (b) and increased precipitation (c) conditions based on Bray–Curtis dissimilarity. The logarithmic between-year similarity values at each plot were fitted to an LMM. The lines show the fixed effects in the LMM. TDR values (*v*) are presented as a coefficient in the fixed effect ± standard error in the random effect. *R*^2^ values represent the variances explained by the whole LMM model. Permutation tests indicated that only the TDR values (*v*) of microbial functional gene community were significantly lower (*P* < .05) under increase precipitation compared to the control.

The observed alterations in soil variables and key ecosystem processes in response to increased precipitation are expected to be accompanied with significant changes in soil microbial communities over time. Permutational multivariate analysis (Adonis) revealed that overall taxonomic and functional structures of soil microbial communities across seven years were different (*P <* .05) between increased precipitation and control (Supplementary Table S2). Moreover, the microbial functional community based on GeoChip measurements was altered by increased precipitation along with the time (NMDS ordination based on the Bray−Curtis and Sorenson metrics; Fig. 2a and Supplementary Fig. S3a). The samples under increased precipitation were gradually separated from the control samples, especially in 2016. However, such a trend was not observed in the taxonomic structures of the prokaryotic and fungal communities (NMDS, Fig. 2a and Supplementary Fig. S3a). Similarly, Adonis analysis in each year revealed that increased precipitation significantly (*P <* .05) shifted soil microbial functional structure in most of the years except 2011 and 2014 (Supplementary Table S3). However, the effects of increased precipitation on prokaryotic communities became significant (*P <* .05) in the last year (2016), and no significant effects were observed on fungal communities in any years (Supplementary Table S3).

Further comparison of the microbial taxonomic composition showed that over the 7 years, the relative abundances of four prokaryotic phyla, including *Euryarchaeota, Bacteroidetes, Chloroflexi*, and *Nitrospirae*, and one fungal phylum *Chytridiomycota* were increased (*P <* .05) by increased precipitation; whereas prokaryotic phyla *Actinobacteria* and *Verrucomicrobia* were decreased (*P <* .05, Supplementary Table S4). However, different prokaryotic/fungal phyla were shifted (*P <* .05) by increased precipitation each year, and no obvious trend was observed for neither prokaryotic nor fungal phyla (Supplementary Fig. S4 and Supplementary Table S5). Specifically, the relative abundances of *Deltaproteobacteria* and *Parcubacteria* were significantly increased in 2012, and *Chloroflexi* and *Planctomycetes* were significantly increased in 2016, while *Verrucomicrobia* in 2016 was significantly decreased. Similarly, increased precipitation led to significant increase in the relative abundances of the fungal phyla *Ascomycota* in 2014 and *Chytridiomycota* in 2015.

To understand the impacts of increased precipitation on the temporal succession of soil microbial community, we calculated TDRs of soil prokaryotes, fungi, and functional genes, using the slope of the linear mixed-effect regression, TDR value (*v*), as the temporal succession rate [20]. There were no significant TDRs under either increased precipitation or control conditions for prokaryotes with abundance-based Bray–Curtis metrics (*v* = 0.026–0.039, *P >* .10; Fig. 2b and c). Significant TDRs were observed for prokaryotes (*v* = 0.026–0.029, *P <* .05) and fungi (*v* = 0.107–0.135, *P <* .01) under both increased precipitation and control conditions based on Sorenson metrics (Supplementary Fig. S2b and c). However, the TDR values (*v*) of neither prokaryotes nor fungi were significantly different between increased precipitation and the control based on permutation tests (*P >* .10), suggesting that the temporal succession rates of microbial taxonomic compositions were not affected by increased precipitation. For microbial functional genes, significant TDRs were observed under both control (*v* = 0.024–0.039, *P <* .01) and increased precipitation (*v* = 0.017–0.025, *P <* .05) conditions based on both Bray–Curtis and Sorenson metrics (Fig. 2b and c and Supplementary Fig. S3b and c). The slopes of TDRs were also significantly or marginally steeper under control than increased precipitation as indicated by permutation tests (*P <* .05 for Bray–Curtis dissimilarity metric, and *P <* .10 for Sorensen dissimilarity metric), suggesting that the temporal succession rates of the microbial functional community were decelerated by increased precipitation (Fig. 2b and c and Supplementary Fig. S3b and c).

Significant (*P <* .05) TDRs were detected in only a few microbial lineages under either control or increased precipitation based on Bray–Curtis and Sorensen metrics (Supplementary Fig. S5, and Supplementary Tables S6, S7), explaining the absence of significant TDRs at the whole-community level (Fig. 2b and c and Supplementary Fig. S3b and c). Specifically, *Chloroflexi, Firmicutes*, and *Zygomycota* only exhibited significant (*P <* .05) TDRs under increased precipitation based on Bray–Curtis metrics, with values higher than those of the control (*P <* .05). In contrast, *Gammaproteobacteria* exhibited significant (*P =* .027) TDRs only in the control based on Bray–Curtis metrics, with values higher than those under increased precipitation (*P =* .043; Supplementary Fig. S5, and Supplementary Table S6). Under Sorensen metrics, although *Actinobacteria, Alphaproteobacteria, Gemmatimonadetes, Nitrospirae*, and *Ascomycota, Basidiomycota* exhibited significant (*P <* .05) TDRs under control and/or increased precipitation, their TDR values did not differ (*P >* .10) between treatments (Supplementary Fig. S5, and Supplementary Table S7). At the functional gene level, subdivision analyses revealed significant (*P <* .05) TDRs for C degradation and nutrient cycling genes under both control and increased precipitation treatments based on Bray–Curtis and Sorensen metrics. Bray–Curtis dissimilarity metric captured a significant (*P <* .05) deceleration in the temporal succession of genes involved in labile C degradation (e.g. hemicellulose, cellulose) and N cycling, whereas Sorensen dissimilarity metric revealed a significant (*P <* .05) deceleration for genes associated with recalcitrant C degradation (e.g. terpenes, vanillin, lignin) and S cycling (Supplementary Fig. S6, and Supplementary Tables S6, S7).

### Effects of increased precipitation on microbial functional genes

**Figure 3:**
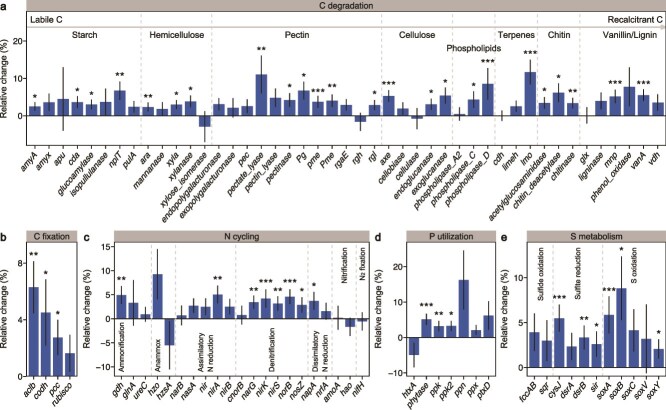
Effects of increased precipitation on microbial functional genes involved in biogeochemical cycling processes across seven years. (a) C degradation genes, arranged by substrate complexity from labile to recalcitrant C. (b) C fixation genes. (c) N cycling processes. (d) P utilization genes. (e) S metabolism processes. The effect size is presented as the average relative change in normalized signal intensity of the detected genes under increased precipitation compared to the control over the seven-year period. Error bars represent standard errors of the relative changes. Significance was assessed using repeated measures ANOVA as indicated by ^***^P < .001, ^**^P < .01, ^*^P < .05.

It is expected that the impacts of increased precipitation on the temporal succession of soil microbial functional community would lead to changes in microbially mediated geochemical cycling processes (e.g. C decomposition and nutrient cycling processes). Therefore, we further analyzed key functional genes involved in C decomposition and nutrient (N, P, and S) cycling processes based on GeoChip measurements of all soil samples collected from 2009 to 2016. All of the eight functional gene groups involved in degrading various C compounds were significantly correlated with *R*_h_ (*P <* .05 by Mantel test, with the exception of pectin, Supplementary Table S8), suggesting that changes in abundance of these C degradation genes could be important in mediating *R*_h_. Specifically, the intensities of >50% of the detected C degradation genes were significantly enhanced (*P <* .05) by increased precipitation across the seven years (Fig. 3a), and all of these significantly increased genes were correlated with *R*_h_ (*P <* .10 by Mantel test, Supplementary Table S9). The increased intensities of genes involved in C degradation could enhance soil C decomposition capacity, consistent with the overall increase in *R*_h_ (Fig. 1g) and the higher soil C decomposition rate observed in the treatment plots in the final year (Fig. 1h), compared to the controls. Moreover, the significantly increased C degradation genes were involved in degrading both liable C (e.g. starch, hemicellulose, and cellulose) and recalcitrant C (e.g. chitin, vanillin, and lignin) (Fig. 3a). In addition to the C degradation genes, the intensity of key genes encoding enzymes for major CO_2_ fixation pathways, including *aclb* in the reverse tricarboxylic acid cycle, *codh* in the reductive acetyl-CoA pathway, and *pcc* for the 3-hydroxypropionate cycle, also increased across seven years (*P <* .05; Fig. 3b), implying that CO_2_ fixation potential was also enhanced by increased precipitation.

The increased precipitation treatment also increased the intensities of genes involved in nutrient cycling processes, including N cycling, P utilization, and S metabolism (*P <* .05; Fig. 3c–e). The intensity of *gdh*, a key gene involved in N mineralization, was higher in increased precipitation than control plots (*P <* .05; Fig. 3c). Also, increased precipitation increased the intensities of several N cycling genes involved in the reductive processes that use nitrate as the electron acceptor, including *nirA* involved in assimilatory nitrate reduction, *narG, nirK, nirS, norB*, and *nosZ* associated with the denitrification process, and *napA*, a reductase involved in the dissimilarity nitrate reduction process (*P <* .05; Fig. 3c). The combined effect of denitrification, assimilatory nitrate reduction, and dissimilatory nitrate reduction could result in a rapid soil NO_3_^−^ loss, consistent with the decreased soil NO_3_^−^ under increased precipitation (Fig. 1c). Moreover, three P utilization genes, including *phytase, ppk*, and *ppk2*, and six S metabolic genes, including *cysJ, dsrB*, and *sir* involved in sulfite reduction, and *soxA, soxB, soxY* mediating S oxidation had higher intensities in the treatment plots than in the control plots (*P <* .05, Fig. 3d and e). The significant increase in abundance of the genes involved in nutrient cycling processes observed in the treatment plots may enhance the rates of soil nutrient cycling and promote plant growth [40], consistent with the increase of GPP in the treatment plots compared to the control plots (Fig. 1f). Furthermore, GPP was correlated with the detected functional gene groups/genes involved in N, P, and S cycling processes (*P <* .10 by Mantel test, Supplementary Tables S8 and S9).

The increased precipitation treatment may have induced temporal shifts in microbial functional dynamics. To assess whether such directional changes occurred, we detected the correlation between gene functional intensities and sampling years. The intensities of genes associated with labile C degradation (including *apu, pme*, and *cellulase*), and the *gdh* gene involved in ammonification within the N cycling exhibited significant correlations with sampling years (adjusted *P <* .05) under increased precipitation, but not in the control group (Supplementary Fig. S7).

### Linking soil microbial communities with environmental variables

**Figure 4:**
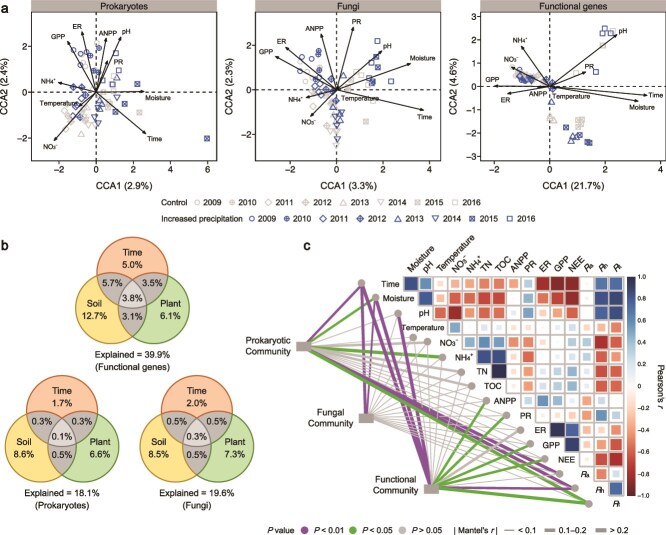
Environmental drivers of the changes in microbial taxonomic and functional community structures. (a) CCA ordinations based on 16S rRNA gene sequencing, ITS gene sequencing, and GeoChip data, illustrating that the community structures of soil prokaryotes, fungi, and functional genes were shaped by soil-related variables: soil moisture, soil pH, soil temperature, soil nitrate (NO_3_^−^), and ammonia (NH_4_^+^); by plant-related variables: plant richness (PR), ANPP, ER, and GPP; and by time. (b) CCA-based VPA showing relative contributions of soil-related variables, plant-related variables, and time to the variations of microbial communities based on Bray–Curtis dissimilarity metric. (c) Mantel tests assessing correlations between microbial taxonomic and functional community composition, and time, soil-related variables, plant-related variables, and ecosystem C fluxes. Edge width corresponds to the Mantel's *r* value, and edge color denotes the statistical significance. Pairwise comparisons of environmental variables with a color gradient denoting Pearson's correlation coefficients. NEE: net ecosystem C exchange. *R*_h_: Soil heterotrophic respiration. *R*_a_: Soil autotrophic respiration. *R*_t_: Soil total respiration.

The weakened succession of the soil microbial functional community structure could be caused by changes in various environmental variables induced by increased precipitation. CCAs showed that the soil microbial functional community structure was shaped by time and several soil- and plant-related environmental variables, including soil moisture, soil pH, NO_3_^−^, and NH_4_^+^ (*P <* .05, Fig. 4a and Supplementary Table S10). A partial CCA-based VPA indicated that as much as 39.9% of the variation in the microbial functional community was explained by soil variables (12.7%), plant variables (6.1%), time (5.0%), and their joint effects (16.1%; Fig. 4b). Both prokaryotic community and fungal community were also correlated with time and other observed environmental variables, such as soil pH (*P <* .05, Supplementary Table S10). However, the first two CCA axes explained only 5.3% of the variation of the prokaryotic community, and 5.6% of the variation of the fungal community, which was much smaller than the explanation of the variation of the functional community structure (26.3%) (Fig. 4a). Additionally, a partial CCA-based VPA analysis indicated that these observed soil variables, plant variables, and time could explain only 18.1% of the variation of the prokaryotic community, and 19.6% of the variation of the fungal community (Fig. 4b).

The measured environmental variables were significantly correlated with the microbial functional community structure (*r* = 0.12, *P =* .031 by Mantel test), but not with the taxonomic structures of the prokaryotic (*r* = −0.02, *P =* .573) or fungal (*r* = 0.04, *P =* .245) communities. More environmental variables exhibited significant (*P <* .05) correlations with the microbial functional community (nine variables) than with the prokaryotic community (six variables) and the fungal community (two variables) (Fig. 4c). The variation of microbial functional community structure was significantly correlated (*P <* .05) with ecosystem C fluxes (e.g. GPP and NEE) and soil respiration (e.g. *R*_h_ and *R*_t_) (Fig. 4c), suggesting that the shifts of microbial functional community structure under increased precipitation could affect soil C cycling processes.

**Figure 5:**
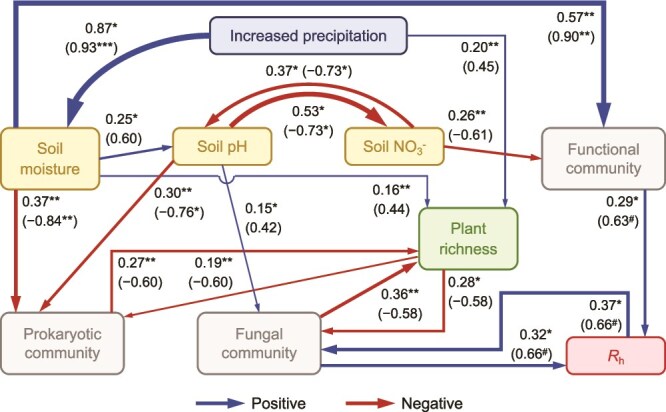
PLS illustrating the effects of increased precipitation treatment on soil physicochemical properties, above-ground plant richness, microbial taxonomic and functional communities, and soil heterotrophic respiration (*R*_h_). In all forward-selected PLS models (adjusted *P* < .05 for both *R*^2^_Y_ and *Q*^2^_Y_; two-sided), the direction of arrows indicates relationships from the independent variable(s) to a dependent variable. The numbers adjacent to the pathway arrows represent the proportion of variance explained for each dependent variable, with the top row representing the partial *R*^2^ index based on PLS (see Methods for details) and the bottom row representing Pearson correlation coefficient (*r*). Asterisks indicate significance levels for the optimal PLS model (upper row) and Pearson correlation (lower row). Blue lines represent positive correlations. Red lines represent negative correlations. Pathway width is scaled proportionally to the partial *R*^2^ value. ^***^P < .001, ^**^P < .01, ^*^P < .05, ^#^*P* < .10.

PLS modeling revealed that increased precipitation exerted a pronounced positive effect on soil moisture (Pearson correlation *r* = 0.93, partial *R*^2^ = 0.87, *P* < .05 based on the PLS model; Fig. 5). In turn, soil moisture directly influenced the soil prokaryotic community (*r* = −0.84, partial *R*^2^ = 0.37, *P* < .01), and exerted indirect effects on both prokaryotic and fungal communities via shifts in soil pH and above-ground plant richness (*P <* .05; Fig. 5). A strong positive correlation was observed between soil moisture and the microbial functional community (*r* = 0.90, partial *R*^2^ = 0.57, *P* < .01), with additional weak positive indirect effects mediated through changes in soil pH and NO_3_^−^ (partial *R*^2^ = 0.26, *P* < .01; Fig. 5). Moreover, soil *R*_h_ was positively affected by soil moisture-induced shifts in soil fungal community (*r* = 0.66, partial *R*^2^ = 0.32, *P* < .05), and microbial functional community (*r* = 0.63, partial *R*^2^ = 0.29, *P* < .05; Fig. 5).

### Assembly mechanisms of soil microbial communities

To quantify the relative roles of stochastic and deterministic processes in shaping soil microbial communities under increased precipitation and control, MST was calculated based on Bray–Curtis and Sorensen dissimilarity metrics [37, 38]. Across the 7 years, the average contributions of stochastic processes to the community variations were 84.4% to 85.1% for prokaryotes, 21.6% to 25.7% for fungi, and 70.7% to 71.9% for microbial functional genes based on Bray–Curtis dissimilarity metric; whereas the portions based on Sorensen dissimilarity metric were 80.0% to 85.0%, 81.9% to 82.1%, and 31.8% to 44.4% (Supplementary Fig. S8). The discrepancy between stochasticity derived from Bray–Curtis and Sorensen dissimilarity metrics could reflect different community assembly mechanisms governing abundant and rare microbial taxa. Furthermore, increased precipitation enlarged the average relative importance of stochastic processes in shaping the functional structure of microbial communities, as quantified using the Sorensen dissimilarity metric, with an observed increase of 39.7% (*P <* .001; Supplementary Fig. S8). Consistently, the contribution of stochastic processes to the assembly of prokaryotic community structure based on Sorensen metrics was higher in treatment plots (85.0%) compared to control plots (80.0%, *P <* .001; Supplementary Fig. S8).

## Discussion

Grassland ecosystems are severely impacted by precipitation regimes, such as intensified rainfall events and/or drought [6]. Until now, limited information is available on the successional dynamics of soil microbial communities and their mediation of C cycle feedbacks to increased precipitation due to the scarcity of time-series data. In this study, we examined the taxonomic and functional succession of the soil microbial communities based on annually collected soil samples over 7 years. Our results indicated that long-term increased precipitation decelerated the temporal succession of soil microbial functional community and stimulated microbial functional genes involved in C decomposition and nutrient cycling, accompanied by larger roles of stochastic processes in shaping soil microbial functional community structure.

Soil microbial communities, which are structurally complex, are sensitive to environmental changes [19]. Compared to snapshot measurements, studies of temporal changes provide more valuable information on the responses of soil microbial communities to climate change and the resulting feedbacks on ecosystem function [41]. A soil transplant study showed that climate warming caused higher soil microbial temporal turnover and less stability, which could be related to increased microbial internal competition [42]. Our previous study also observed significantly accelerated temporal turnover rates of soil microbial communities under warming, and warming significantly decreased the relative importance of stochastic processes in shaping microbial communities, indicating the significant selection on soil microorganisms imposed by warming [20, 35]. Considering the critical roles of water availability (either as precipitation or soil moisture) in water-limited grassland ecosystems [22], increased precipitation, contrary to warming, may alleviate environmental stress with more water availability. As a result, the temporal succession of soil microbial functional community structure was significantly decreased due to increased precipitation in our study. Consistently, the relative importance of stochastic processes in shaping soil microbial functional community was significantly increased by increased precipitation, indicating that the effects of the deterministic filtering factors in the environment that imposed selection on microorganisms were weakened due to more water availability, which, as expected, is opposite to the effects of warming on community assembly. Additionally, the contrasting patterns of significant TDRs between microbial taxonomic and functional communities, as revealed by Bray–Curtis and Sorensen metrics (Fig. 2 and Supplementary Fig. S3), suggest that taxonomic succession is primarily driven by the replacement of rare taxa [43, 44], as significant TDRs were detected only under the Sorensen metric. In contrast, functional community succession appears to be influenced mainly by fluctuations in the intensity of abundant genes, given that Bray–Curtis values were consistently higher than Sorensen values [43, 44] (Fig. 2 and Supplementary Fig. S3).

Our study revealed a decoupling in the temporal succession of soil microbial taxonomic and functional community structures in response to increased precipitation, extending the framework that microbial taxonomy and function can vary independently along temporal dimensions [45, 46]. This decoupling likely arises from different mechanisms shaping taxonomic and functional communities: phylogenetically distant lineages may share similar functions, whereas closely related taxa can fulfill divergent metabolic roles [47]. Additionally, the higher dimensionality of functional profiles, given that microbial genomes harbor thousands of functional genes, may further render functional communities more sensitive to increased precipitation. However, methodological discrepancies warrant consideration, as GeoChip offers higher detection resolution and provides reliable quantitative information on targeted genes compared to amplicon-based taxonomic profiling [48]. Future studies employing unified analytical frameworks are needed to validate this decoupling.

As soil microbial communities play important roles in the biogeochemical cycling of C, N, P, and S, elucidating the effects of increased precipitation on the temporal succession of microbial community functions is essential for robustly predicting soil C fluxes and storage. To our knowledge, however, only a few studies investigated the microbial functional responses to increased precipitation by measuring the activities of several key enzymes involved in soil C decomposition and nutrient cycling, and increased enzyme activities were typically observed in these studies [10, 12]. Based on annually collected samples and GeoChip analysis, we demonstrated that increased precipitation treatment significantly increased the abundances of multiple genes involved in C decomposition and nutrient cycling. We further provided evidence that the enhanced gene abundances were closely linked to increased precipitation-induced changes in environmental variables (e.g. soil moisture, pH, and ANPP), and were positively associated with ecosystem functions such as *R*_h_, with potential implications for the stability of grassland C storage.

In conclusion, the temporal succession of microbial communities is a crucial issue in global change biology. However, relevant studies are scarce, mainly due to the lack of time-series data under long-term climate change experiments. Here, we documented that increased precipitation weakened the temporal succession of soil microbial functional community structure but not taxonomic community structure. The decoupling between microbial functional and taxonomic succession in response to increased precipitation challenges the reliance on taxonomic metrics alone, and highlights the need to integrate functional responses into predictive models of soil feedbacks to climate change [9, 40]. Moreover, increased precipitation stimulated the abundance of microbial functional genes for C decomposition, which positively correlated with the enhanced *R*_h_, likely resulting from an increased microbial decomposition potential of soil C. Soil nutrient cycling processes were potentially enhanced by increased precipitation, which could promote plant nutrient use efficiency and increase plant growth (e.g. GPP). However, increased precipitation strengthened the importance of stochastic processes in shaping microbial communities, implying that microbial successional trajectories may shift toward multiple alternative states characterized by greater stochasticity under future altered precipitation scenarios.

## Supplementary Material

Supplemental_information_ISME_R2_wrag176

## Data Availability

DNA sequence data of 16S rRNA gene and ITS amplicons are available in NCBI Sequence Read Archive under BioProject accession number PRJNA331185. Source data, including environmental variables, OTU tables, GeoChip signal intensity data, and all R scripts used for statistical analyses, are publicly available on GitHub at https://github.com/Deng-Sihang/wetting.
