# Supplementary material for: Increased precipitation decelerates temporal succession of grassland soil microbial communities
Source: ISME J. 2026 Jul 12;20(1):wrag176. doi: 10.1093/ismejo/wrag176 (PMC13440302; doi:10.1093/ismejo/wrag176)
Supplement: Supplementary file 1 [file supplemental_information_isme_r2_wrag176.docx]

**SUPPLEMENTARY INFORMATION** for

**Increased precipitation decelerates temporal succession of grassland soil microbial communities**

Sihang Deng, Weiling Shi, Renmao Tian, Xue Guo, Daliang Ning, Xishu Zhou, Mengting Yuan, Jiajie Feng, Aifen Zhou, Ying Fu, Gang Xu, Dashuai Mu, Peng Shi, Xiangyu Fan, Yue Teng, Xin Zhao, Zhongfang Li, Jiantao Liu, Xiaonan Liu, Liyou Wu, Zhili He, Xueduan Liu, Yiqi Luo, James M. Tiedje, Yunfeng Yang, and Jizhong Zhou

Address correspondence to Jizhong Zhou, jzhou@ou.edu

**This file includes:**

Figures S1 to S8

Tables S1 to S10

**Fig. S1. Schematic diagram of the field experimental treatments.** In this long-term climate change experiment, warming (+3 °C), half precipitation (−50%), and double precipitation (+100%) are primary factors, which are nested with clipping as the secondary factor (all the 24 southern subplots are under clipping treatment). Thus, this experiment has twelve single and combined treatments as follows: Control (N), Warming (W), Half precipitation (H), Double precipitation (D), Clipping (C), Warming & Half precipitation (WH), Warming & Double precipitation (WD), Warming & Clipping (WC), Half precipitation & Clipping (HC), Double precipitation & Clipping (DC), Warming & Half precipitation & Clipping (WHC), and Warming & Double precipitation & Clipping (WDC). Each treatment is replicated four times across four different blocks. The present study focuses on the control (N) and double precipitation (D) treatments, which are indicated by red boxes.

**Fig. S2.** **Effects of increased precipitation (IP) on soil variables and ecosystem C fluxes across seven years.** (**a**) Soil temperature. (**b**) Soil ammonium (NH_4_^+^). (**c**) Soil total organic carbon (TOC). (**d**) Total plant biomass. (**e**) C_3_ plant biomass. (**f**) C_4_ plant biomass. (**g**) Plant richness. (**h**) Soil autotrophic respiration (*R*_a_). (**i**) Soil total respiration (*R*_t_). (**j**) Net ecosystem exchange (NEE). Year 0 represents the year 2009. Error bars indicate standard error. The differences between increased precipitation (blue bar) and the control (grey bar) for each year and across all years were assessed using repeated measures ANOVA. Significance levels denoted as *** *P <* 0.001, ** *P <* 0.01, * *P <* 0.05, ^#^ *P <* 0.10. No significant interaction effects between treatment and sampling year were detected for the measured soil variables or ecosystem C fluxes, with the exception of NEE, for which the interaction was significant (*P* = 0.045).

**Fig. S3. Responses of soil microbial taxonomic and functional communities to increased precipitation over time based on Sorenson dissimilarity**. (**a**) Non-metric multidimensional scaling (NMDS) ordination illustrating temporal changes in prokaryotic (16S rRNA gene sequencing), fungal (ITS sequencing), and functional gene (GeoChip) communities under control and increased precipitation conditions. In the first year (2009), samples from both treatments clustered together (encircled in black). In subsequent years, particularly evident in 2016, communities under increased precipitation progressively separated from controls, with the most pronounced separation observed for the functional gene community (blue circle: increased precipitation; grey circle: control). The time-decay relationships (TDRs) for prokaryotic, fungal, and functional gene communities under control (**b**) and increased precipitation (**c**) conditions. The logarithmic between-year similarity values at each plot were fitted to a linear mixed model (LMM). The lines show the fixed effects in the LMM. TDR values (*v*) are presented as a coefficient in the fixed effect ± standard error in the random effect. *R*^2^ values represent the variances explained by the whole LMM model. Permutation tests indicated that only the TDR values (*v*) of microbial functional gene community were marginally lower (*P <* 0.10) under increase precipitation compared to the control.

**Fig. S4.** **Prokaryotic and fungal community composition under control and increased precipitation (IP) conditions over seven years.** The phylum *Proteobacteria* was represented by Alpha-, Beta-, Gamma-, and Delta- divisions.

**Fig. S5. Time-decay relationships (TDRs) for different prokaryotic and fungal lineages under control and increased precipitation conditions.** (**a**) Bray-Curtis dissimilarity. (**b**) Sorenson dissimilarity. TDR values (*v*) are presented as fixed-effect coefficients ± standard errors from linear mixed-effects models. Filled circles indicate significant TDRs (*P* < 0.05); open circles indicate non-significant TDRs. Blue circles represent increased precipitation, grey circles represent control. Permutation tests were used to compare TDR values between treatments. Significance levels denoted as ** *P <* 0.01, * *P <* 0.05. See Supplementary Tables 6 and 7 for detailed statistics.

**Fig. S6. Time-decay relationships (TDRs) for different microbial functional genes under control and increased precipitation conditions.** (**a**) Bray-Curtis dissimilarity. (**b**) Sorenson dissimilarity. TDR values (*v*) are presented as fixed-effect coefficients ± standard errors from linear mixed-effects models. Filled circles indicate significant TDRs (*P* < 0.05); open circles indicate non-significant TDRs. Blue circles represent increased precipitation, grey circles represent control. Permutation tests were used to compare TDR values between treatments. Significance levels denoted as *** *P <* 0.001, ** *P <* 0.01, * *P <* 0.05, ^#^ *P <* 0.10. See Supplementary Tables 6 and 7 for detailed statistics.

**Fig. S7. Temporal dynamics of microbial functional genes in response to increased precipitation over the seven-year period.** C degradation genes: (**a**) *apu*, (**b**) *pme*, (**c**) *cellulase*. (**d**) *gdh*, involved in ammonification within N cycling. The slopes were estimated using linear mixed-effects models (LMMs) accounting for the repeated-measure design. *R*^2^ values represent the variances explained by the whole LMM model. Significance was determined using Wald-type II *χ*^2^ tests (n = 32). The lines show the fixed effects in the LMM. The first year (0) is 2009. The normalized signal intensities of all four genes increased significantly over time under increased precipitation condition (blue lines), whereas no significant temporal trends were observed in the control group (grey lines).

**Fig. S8. Overall stochasticity of microbial taxonomic and functional communities under control and increased precipitation conditions.** Stochasticity was assessed using (**a**) Bray-Curtis and (**b**) Sorensen metrics based on 16S rRNA gene sequencing, ITS gene sequencing, and GeoChip datasets. Error bars indicate standard error. The differences between increased precipitation (blue bar) and the control (grey bar) across seven years were assessed using repeated measures ANOVA. Significance levels denoted as *** *P <* 0.001.

**Table S1. Candidate predictor variables (independent variables, X) considered for each dependent variable (Y) in partial least squares (PLS) modeling. All listed predictors were included in the forward selection procedure, and the optimal model was identified as described in Methods.**

| **No.** | **Dependent variable (Y)** | **Potential predictors (independent variables, X, in PLS)**^[1]^ |
| --- | --- | --- |
| 1 | Soil moisture | Increased precipitation, Soil pH, Soil NO_3_^−^ |
| 2 | Soil pH | Increased precipitation, Soil moisture, Soil NO_3_^−^ |
| 3 | Soil NO_3_^−^ | Increased precipitation, Soil moisture, Soil pH |
| 4 | Plant richness | Increased precipitation, Soil moisture, Soil pH, Soil NO_3_^−^, Prokaryotic community (16S rRNA, NMDS1), Fungal community (ITS, NMDS1), Functional community (Geochip, NMDS1) |
| 5 | Prokaryotic community  (16S rRNA, NMDS1) | Increased precipitation, Soil moisture, Soil pH, Soil NO_3_^−^, Plant richness, Fungal community (ITS, NMDS1), Functional community (Geochip, NMDS1), *R*_h_ |
| 6 | Fungal community  (ITS, NMDS1) | Increased precipitation, Soil moisture, Soil pH, Soil NO_3_^−^, Plant richness, Prokaryotic community (16S rRNA, NMDS1), Functional community (Geochip, NMDS1), *R*_h_ |
| 7 | Functional community  (Geochip, NMDS1) | Increased precipitation, Soil moisture, Soil pH, Soil NO_3_^−^, Plant richness, Prokaryotic community (16S rRNA, NMDS1), Fungal community (ITS, NMDS1), *R*_h_ |
| 8 | *R*_h_ | Increased precipitation, Soil moisture, Soil pH, Soil NO_3_^−^, Plant richness, Prokaryotic community (16S rRNA, NMDS1), Fungal community (ITS, NMDS1), Functional community (Geochip, NMDS1) |

[1] Microbial taxonomic and functional communities were represented by the first axis of non-metric multidimensional scaling (NMDS1) derived from Bray-Curtis dissimilarity metrics.

**Table S2. Summary of permutational multivariate analysis of increased precipitation (IP), year, and block on microbial communities.**

| **Datasets** | **Prokaryotes (16S rRNA)** | | | | **Fungi (ITS)** | | | | **Functional genes (GeoChip)** | | | |
| --- | --- | --- | --- | --- | --- | --- | --- | --- | --- | --- | --- | --- |
| **Items**^[1]^ | ***Df*** | ***F*** | ***R*^2^** | ***P***^[2]^ | ***Df*** | ***F*** | ***R*^2^** | ***P*** | ***Df*** | ***F*** | ***R*^2^** | ***P*** |
| IP | 1 | 1.844 | 0.02 | **0.009** | 1 | 1.311 | 0.02 | **0.049** | 1 | 4.803 | 0.03 | **0.005** |
| Year | 7 | 2.639 | 0.24 | **0.001** | 7 | 1.983 | 0.20 | **0.001** | 7 | 15.28 | 0.56 | **0.001** |
| Block | 3 | 1.516 | 0.06 | **0.003** | 3 | 1.339 | 0.06 | **0.008** | 3 | 1.805 | 0.03 | **0.034** |
| IP × Year | 7 | 1.077 | 0.10 | 0.199 | 7 | 0.990 | 0.10 | 0.534 | 7 | 3.908 | 0.14 | **0.001** |
| Residuals | 45 | - | 0.58 | - | 45 | - | 0.62 | - | 45 | - | 0.24 | - |

[1] The increased precipitation effects on microbial taxonomic and functional communities were evaluated using the permutational multivariate analysis of variance (Adonis), setting as “Dissimilarity ~ IP × Year + Block” based on Bray–Curtis dissimilarities among samples.
[2] Significant effects (*P <* 0.05) are shown in bold.

**Table S3.** **Summary of permutational multivariate analysis of increased precipitation (IP) on microbial communities in each year.**

| **Datasets** | **Prokaryotes (16S rRNA)** | | **Fungi (ITS)** | | **Functional genes (GeoChip)** | |
| --- | --- | --- | --- | --- | --- | --- |
| **Year**^[1]^ | ***R*^2^** | ***P***^[2]^ | ***R*^2^** | ***P*** | ***R*^2^** | ***P*** |
| 2009 | 0.14 | 0.519 | 0.15 | 0.052 | 0.27 | **0.043** |
| 2010 | 0.18 | 0.079 | 0.16 | 0.191 | 0.25 | **0.007** |
| 2011 | 0.14 | 0.633 | 0.14 | 0.308 | 0.20 | 0.131 |
| 2012 | 0.14 | 0.290 | 0.13 | 0.482 | 0.21 | **0.002** |
| 2013 | 0.15 | 0.145 | 0.11 | 0.681 | 0.32 | **0.002** |
| 2014 | 0.16 | 0.512 | 0.16 | 0.276 | 0.19 | 0.092 |
| 2015 | 0.15 | 0.488 | 0.16 | 0.301 | 0.50 | **0.032** |
| 2016 | 0.23 | **0.015** | 0.13 | 0.692 | 0.56 | **0.034** |

[1] The increased precipitation effects on microbial taxonomic and functional communities were evaluated using the permutational multivariate analysis of variance (Adonis), setting as “Dissimilarity ~ IP + Block” based on Bray–Curtis dissimilarities among samples.
[2] Significant effects (*P <* 0.05) are shown in bold.

**Table S****4. ANOVA results for the effects of increased precipitation and sampling year on the relative abundances of prokaryotic and fungal phyla over seven years.**

| **Phylum**^[1]^ | **Relative abundance**^[2]^ | | **IP**^[3]^ | | **Year** | |
| --- | --- | --- | --- | --- | --- | --- |
|  | **Control** | **IP** | ***F*** | ***P***^[4]^ | ***F*** | ***P*** |
| **Prokaryotic (Archaea)** |  |  |  |  |  |  |
| Euryarchaeota | **0.04 ± 0.01%** | **0.07 ± 0.01%** | **4.706** | **0.035** | **2.978** | **0.012** |
| Thaumarchaeota | 1.1 ± 0.1% | 1.3 ± 0.1% | 3.037 | 0.088 | **3.374** | **0.006** |
| **Prokaryotic (Bacteria)** |  |  |  |  |  |  |
| Acidobacteria | 16.5 ± 0.6% | 17.1 ± 0.5% | 0.726 | 0.399 | 2.208 | 0.051 |
| Actinobacteria | **16.4 ± 0.8%** | **14.8 ± 0.7%** | **4.627** | **0.037** | **10.19** | **< 0.001** |
| Alphaproteobacteria | 16.2 ± 0.5% | 15.6 ± 0.4% | 1.060 | 0.309 | 1.762 | 0.119 |
| Armatimonadetes | 0.3 ± 0.02% | 0.3 ± 0.02% | 0.015 | 0.903 | **2.911** | **0.013** |
| Bacteroidetes | **4.2 ± 0.3%** | **5.3 ± 0.5%** | **4.781** | **0.034** | **3.614** | **0.004** |
| Betaproteobacteria | 4.2 ± 0.3% | 4.6 ± 0.3% | 1.278 | 0.264 | **3.443** | **0.005** |
| Chlamydiae | 0.8 ± 0.1% | 0.8 ± 0.1% | 1.700 | 0.199 | **11.65** | **< 0.001** |
| Chloroflexi | **0.8 ± 0.03%** | **0.9 ± 0.04%** | **6.303** | **0.016** | **5.523** | **< 0.001** |
| Deltaproteobacteria | 3.4 ± 0.1% | 3.7 ± 0.2% | 2.727 | 0.106 | **4.909** | **< 0.001** |
| Firmicutes | 9.4 ± 1.0% | 8.7 ± 0.8% | 0.334 | 0.566 | **2.343** | **0.040** |
| Gammaproteobacteria | 3.4 ± 0.3% | 4.2 ± 0.5% | 2.702 | 0.107 | **2.544** | **0.027** |
| Gemmatimonadetes | 1.7 ± 0.1% | 1.6 ± 0.1% | 1.254 | 0.269 | **7.869** | **< 0.001** |
| Latescibacteria | 0.1 ± 0.01% | 0.1 ± 0.02% | 3.707 | 0.061 | **2.898** | **0.014** |
| Nitrospirae | **0.6 ± 0.04%** | **0.7 ± 0.04%** | **8.079** | **0.007** | **3.470** | **0.005** |
| Parcubacteria | 0.1 ± 0.01% | 0.1 ± 0.01% | 3.787 | 0.058 | **4.610** | **< 0.001** |
| Planctomycetes | 2.4 ± 0.1% | 2.5 ± 0.1% | 0.019 | 0.891 | **4.275** | **0.001** |
| Verrucomicrobia | **10.0 ± 0.7%** | **8.2 ± 0.3%** | **4.685** | **0.036** | 1.142 | 0.355 |
| **Fungi** |  |  |  |  |  |  |
| Ascomycota | 71.3 ± 3.0% | 71.0 ± 3.5% | 0.005 | 0.944 | 1.792 | 0.112 |
| Basidiomycota | 23.5 ± 3.0% | 19.8 ± 2.9% | 0.809 | 0.373 | 1.194 | 0.326 |
| Chytridiomycota | **0.1 ± 0.02%** | **0.2 ± 0.03%** | **4.684** | **0.036** | **2.822** | **0.016** |
| Glomeromycota | 1.0 ± 0.3% | 0.9 ± 0.3% | 0.009 | 0.927 | **2.440** | **0.033** |
| Zygomycota | 2.6 ± 0.6% | 3.0 ± 0.6% | 0.480 | 0.492 | **5.767** | **< 0.001** |

[1] Proteobacteria was analyzed separately at the class level: Alpha-, Beta-, Gamma-, and Deltaproteobacteria.
[2] Relative abundances are presented as: mean ± SE (n = 32 across seven years), with significant differences (*P <* 0.05) between control and increased precipitation (IP) treatment are given in bold.
[3] A two-sided repeated measures ANOVA model was applied: Relative abundance of phylum ~ IP × Year + Block.
[4] Significant effects of increased precipitation and sampling year (*P <* 0.05) are given in bold.

**Table S5. ANOVA results for the effect of increased precipitation on the relative abundance of each prokaryotic and fungal phylum in each year.**

| **Phylum**^[1]^ | **2009** | | **2010** | | **2011** | | **2012** | | **2013** | | **2014** | | **2015** | | **2016** | |
| --- | --- | --- | --- | --- | --- | --- | --- | --- | --- | --- | --- | --- | --- | --- | --- | --- |
|  | ***F*** | ***P***^[2]^ | ***F*** | ***P*** | ***F*** | ***P*** | ***F*** | ***P*** | ***F*** | ***P*** | ***F*** | ***P*** | ***F*** | ***P*** | ***F*** | ***P*** |
| **Prokaryotic (Archaea)** |  |  |  |  |  |  |  |  |  |  |  |  |  |  |  |  |
| Euryarchaeota | 0.005 | 0.949 | 0.313 | 0.615 | 0.017 | 0.905 | 0.995 | 0.392 | 6.005 | 0.092 | 3.426 | 0.161 | 0.212 | 0.677 | 0.475 | 0.540 |
| Thaumarchaeota | 0.028 | 0.877 | 0.733 | 0.455 | 0.203 | 0.683 | 0.201 | 0.684 | 0.003 | 0.957 | 0.526 | 0.521 | 1.822 | 0.270 | 0.114 | 0.758 |
| **Prokaryotic (Bacteria)** |  |  |  |  |  |  |  |  |  |  |  |  |  |  |  |  |
| Acidobacteria | 0.329 | 0.606 | 0.821 | 0.432 | 1.835 | 0.269 | 0.132 | 0.740 | 1.744 | 0.278 | 1.580 | 0.298 | 2.634 | 0.203 | 0.455 | 0.548 |
| Actinobacteria | 2.863 | 0.189 | 0.012 | 0.918 | 0.664 | 0.475 | 0.135 | 0.738 | 2.518 | 0.211 | 0.264 | 0.643 | 3.119 | 0.176 | 0.043 | 0.848 |
| Alphaproteobacteria | 1.854 | 0.267 | 2.207 | 0.234 | 0.967 | 0.398 | 0.010 | 0.926 | 0.329 | 0.606 | 1.697 | 0.284 | 0.082 | 0.793 | 1.392 | 0.323 |
| Armatimonadetes | 0.038 | 0.858 | 1.460 | 0.313 | 0.415 | 0.565 | 0.002 | 0.965 | 2.320 | 0.225 | 2.801 | 0.193 | 0.383 | 0.580 | 0.042 | 0.851 |
| Bacteroidetes | 1.084 | 0.374 | 0.016 | 0.906 | 0.260 | 0.645 | 1.265 | 0.343 | 8.564 | 0.061 | 0.335 | 0.604 | 1.689 | 0.285 | 4.060 | 0.137 |
| Betaproteobacteria | 0.077 | 0.800 | 5.086 | 0.109 | 1.248 | 0.345 | 5.395 | 0.103 | 3.966 | 0.140 | 0.075 | 0.801 | 1.922 | 0.260 | 0.644 | 0.481 |
| Chlamydiae | 0.003 | 0.959 | 0.013 | 0.917 | 0.053 | 0.832 | 0.062 | 0.819 | 0.765 | 0.446 | 0.043 | 0.850 | 3.344 | 0.165 | 0.096 | 0.776 |
| Chloroflexi | 1.308 | 0.336 | 0.620 | 0.488 | 0.063 | 0.817 | 0.048 | 0.840 | 0.374 | 0.584 | 0.683 | 0.469 | 1.744 | 0.278 | **12.78** | **0.037** |
| Deltaproteobacteria | 0.111 | 0.761 | 0.764 | 0.446 | 0.054 | 0.831 | **15.75** | **0.029** | 0.408 | 0.568 | 1.310 | 0.335 | 0.914 | 0.409 | 0.539 | 0.516 |
| Firmicutes | 0.246 | 0.654 | 0.656 | 0.477 | 0.068 | 0.811 | 5.356 | 0.104 | 0.065 | 0.815 | 1.142 | 0.364 | 0.165 | 0.712 | 1.091 | 0.373 |
| Gammaproteobacteria | 1.021 | 0.387 | 0.060 | 0.823 | 0.377 | 0.582 | 2.086 | 0.244 | 5.414 | 0.102 | 1.620 | 0.293 | 1.241 | 0.347 | 2.918 | 0.186 |
| Gemmatimonadetes | 1.443 | 0.316 | 0.845 | 0.426 | 0.000 | 1.000 | 1.891 | 0.263 | 1.914 | 0.261 | 0.511 | 0.526 | 1.591 | 0.296 | 0.930 | 0.406 |
| Latescibacteria | 3.758 | 0.148 | 0.004 | 0.955 | 1.558 | 0.301 | 3.657 | 0.152 | 2.054 | 0.247 | 0.849 | 0.425 | 3.352 | 0.165 | 0.054 | 0.831 |
| Nitrospirae | 1.099 | 0.372 | 0.779 | 0.442 | 0.043 | 0.849 | 0.914 | 0.410 | 4.785 | 0.117 | 0.366 | 0.588 | 0.872 | 0.419 | 1.238 | 0.347 |
| Parcubacteria | 1.028 | 0.385 | 0.000 | 1.000 | 0.904 | 0.412 | **12.77** | **0.037** | 0.183 | 0.698 | 0.339 | 0.601 | 0.077 | 0.800 | 1.384 | 0.324 |
| Planctomycetes | 0.133 | 0.740 | 0.617 | 0.489 | 0.932 | 0.406 | 0.305 | 0.619 | 0.237 | 0.660 | 0.388 | 0.577 | 3.601 | 0.154 | **11.38** | **0.043** |
| Verrucomicrobia | 0.001 | 0.983 | 3.294 | 0.167 | 1.011 | 0.389 | 0.619 | 0.489 | 0.039 | 0.857 | 0.217 | 0.673 | 0.921 | 0.408 | **30.25** | **0.012** |
| **Fungi** |  |  |  |  |  |  |  |  |  |  |  |  |  |  |  |  |
| Ascomycota | 0.002 | 0.968 | 0.503 | 0.529 | 1.322 | 0.334 | 3.238 | 0.170 | 2.964 | 0.184 | **12.85** | **0.037** | 2.552 | 0.208 | 2.343 | 0.223 |
| Basidiomycota | 0.000 | 0.997 | 1.325 | 0.333 | 1.160 | 0.360 | 1.413 | 0.320 | 0.905 | 0.412 | 9.359 | 0.055 | 0.773 | 0.444 | 1.944 | 0.258 |
| Chytridiomycota | 0.082 | 0.794 | 0.103 | 0.769 | 1.555 | 0.301 | 0.477 | 0.539 | 1.568 | 0.299 | 3.212 | 0.171 | **22.64** | **0.018** | 5.698 | 0.097 |
| Glomeromycota | 0.383 | 0.580 | 6.226 | 0.088 | 0.838 | 0.428 | 0.699 | 0.465 | 1.158 | 0.361 | 0.337 | 0.602 | 0.411 | 0.567 | 1.256 | 0.344 |
| Zygomycota | 0.880 | 0.417 | 0.690 | 0.467 | 0.055 | 0.830 | 1.659 | 0.288 | 0.570 | 0.505 | 0.317 | 0.613 | 2.274 | 0.229 | 0.014 | 0.914 |

[1] Proteobacteria was analyzed separately at the class level: Alpha-, Beta-, Gamma-, and Deltaproteobacteria.
[2] ANOVA model was applied: Relative abundance of phylum ~ IP + Block. Significant effects of increased precipitation (IP) treatment (*P <* 0.05) are given in bold.

**Table S6. TDR values (*v*) of different microbial lineages and functional gene groups based on Bray-Curtis dissimilarity metrics.**

| **Class** | **Control** | | | **Increased precipitation (IP)** | | | **C *vs.* IP**^[3]^ |
| --- | --- | --- | --- | --- | --- | --- | --- |
|  | **slope**^[1]^ | ***R^2^*** | ***P***^[2]^ | **slope** | ***R^2^*** | ***P*** |  |
| **Prokaryotic (Archaea)** |  |  |  |  |  |  |  |
| Euryarchaeota | 0.160 ± 0.102 | 0.13 | 0.275 | **0.205 ± 0.071** | **0.19** | **0.047** | 0.392 |
| Thaumarchaeota | 0.026 ± 0.045 | 0.23 | 0.567 | −0.023 ± 0.046 | 0.03 | 0.636 | 0.200 |
| **Prokaryotic (Bacteria)** |  |  |  |  |  |  |  |
| Acidobacteria | 0.012 ± 0.026 | 0.29 | 0.624 | 0.024 ± 0.014 | 0.21 | 0.189 | 0.330 |
| Actinobacteria | 0.041 ± 0.020 | 0.06 | 0.123 | 0.032 ± 0.024 | 0.16 | 0.294 | 0.376 |
| Alphaproteobacteria | 0.018 ± 0.016 | 0.17 | 0.510 | −0.00001 ± 0.020 | 0.13 | 0.998 | 0.220 |
| Armatimonadetes | −0.002 ± 0.057 | 0.21 | 0.971 | −0.002 ± 0.050 | 0.16 | 0.308 | 0.504 |
| Bacteroidetes | 0.014 ± 0.034 | 0.04 | 0.937 | −0.0004 ± 0.047 | 0.13 | 0.930 | 0.379 |
| Betaproteobacteria | 0.030 ± 0.027 | 0.21 | 0.391 | 0.009 ± 0.036 | 0.14 | 0.947 | 0.346 |
| Chlamydiae | 0.010 ± 0.050 | 0.05 | 0.970 | 0.032 ± 0.043 | 0.03 | 0.651 | 0.328 |
| Chloroflexi | −0.007 ± 0.033 | 0.18 | 0.954 | **0.071 ± 0.031** | **0.15** | **0.045** | **0.032** |
| Deltaproteobacteria | 0.044 ± 0.019 | 0.43 | 0.092 | 0.051 ± 0.032 | 0.21 | 0.110 | 0.396 |
| Firmicutes | 0.004 ± 0.056 | 0.25 | 0.563 | **0.131 ± 0.065** | **0.36** | **0.024** | **0.018** |
| Gammaproteobacteria | **0.093 ± 0.036** | **0.09** | **0.027** | 0.014 ± 0.052 | 0.12 | 0.927 | **0.043** |
| Gemmatimonadetes | 0.066 ± 0.031 | 0.20 | 0.099 | **0.068 ± 0.026** | **0.26** | **0.043** | 0.451 |
| Latescibacteria | −0.116 ± 0.114 | 0.01 | 0.392 | 0.082 ± 0.097 | 0.03 | 0.486 | 0.067 |
| Nitrospirae | −0.024 ± 0.034 | 0.08 | 0.737 | 0.008 ± 0.027 | 0.18 | 0.500 | 0.202 |
| Parcubacteria | 0.090 ± 0.104 | 0.20 | 0.409 | 0.139 ± 0.108 | 0.27 | 0.155 | 0.350 |
| Planctomycetes | 0.036 ± 0.026 | 0.23 | 0.372 | 0.004 ± 0.020 | 0.06 | 0.783 | 0.112 |
| Verrucomicrobia | −0.008 ± 0.034 | 0.15 | 0.952 | 0.018 ± 0.019 | 0.06 | 0.532 | 0.257 |
| **Fungi** |  |  |  |  |  |  |  |
| Ascomycota | 0.118 ± 0.058 | 0.22 | 0.092 | 0.076 ± 0.092 | 0.13 | 0.267 | 0.308 |
| Basidiomycota | 0.329 ± 0.167 | 0.10 | 0.074 | **0.464 ± 0.234** | **0.16** | **0.010** | 0.252 |
| Chytridiomycota | 0.021 ± 0.158 | < 0.01 | 0.952 | 0.155 ± 0.220 | 0.23 | 0.479 | 0.304 |
| Glomeromycota | 0.010 ± 0.124 | 0.04 | 0.909 | −0.299 ± 0.262 | 0.26 | 0.254 | 0.074 |
| Zygomycota | −0.030 ± 0.137 | 0.07 | 0.911 | **0.423 ± 0.181** | **0.24** | **0.003** | **0.007** |
| **C degradation** |  |  |  |  |  |  |  |
| Starch | **0.045 ± 0.008** | **0.30** | **0.002** | **0.032 ± 0.005** | **0.26** | **0.006** | 0.091 |
| Hemicellulose | **0.039 ± 0.008** | **0.25** | **0.003** | **0.025 ± 0.005** | **0.26** | **0.009** | **0.044** |
| Pectin | **0.048 ± 0.008** | **0.25** | **0.001** | **0.031 ± 0.006** | **0.26** | **0.005** | 0.094 |
| Cellulose | **0.050 ± 0.010** | **0.32** | **0.003** | **0.037 ± 0.006** | **0.29** | **0.007** | **0.047** |
| Phospholipids | **0.064 ± 0.016** | **0.13** | **0.001** | **0.027 ± 0.008** | **0.16** | **0.024** | 0.217 |
| Terpenes | **0.035 ± 0.006** | **0.31** | **0.003** | **0.030 ± 0.004** | **0.31** | **0.004** | 0.273 |
| Chitin | **0.049 ± 0.009** | **0.24** | **0.003** | **0.028 ± 0.005** | **0.26** | **0.003** | 0.115 |
| Vanillin/Lignin | **0.050 ± 0.009** | **0.29** | **0.005** | **0.041 ± 0.009** | **0.35** | **0.005** | 0.261 |
| **Nutrient cycling genes** |  |  |  |  |  |  |  |
| N cycling | **0.039 ± 0.007** | **0.25** | **0.006** | **0.022 ± 0.004** | **0.23** | **0.009** | **0.029** |
| P utilization | **0.042 ± 0.008** | **0.29** | **< 0.001** | **0.030 ± 0.005** | **0.26** | **0.011** | 0.089 |
| S metabolism | **0.040 ± 0.007** | **0.27** | **0.003** | **0.027 ± 0.005** | **0.24** | **0.012** | 0.119 |

[1] The slopes were estimated using linear mixed−effects models (LMMs). *R*^2^ values represent the variances explained by the whole LMM model. The slopes are presented as a coefficient in fixed effect ± standard error in random effect.
[2] Statistical significance was determined using permutation tests with 1000 randomizations. Significant effects (*P <* 0.05) are indicated in bold.
[3] Statistical significance of the differences in TDR values between the increased precipitation treatment and the control was determined using permutation tests with 1000 randomizations.

**Table S7. TDR values (*v*) of different microbial lineages and functional gene groups based on Sorensen metrics.**

| **Class** | **Control** | | | **Increased precipitation (IP)** | | | **C *vs.* IP**^[3]^ |
| --- | --- | --- | --- | --- | --- | --- | --- |
|  | **slope**^[1]^ | ***R^2^*** | ***P***^[2]^ | **slope** | ***R^2^*** | ***P*** |  |
| **Prokaryotic (Archaea)** |  |  |  |  |  |  |  |
| Euryarchaeota | 0.051 ± 0.080 | 0.13 | 0.793 | 0.102 ± 0.062 | 0.14 | 0.243 | 0.373 |
| Thaumarchaeota | −0.003 ± 0.014 | 0.20 | 0.911 | 0.006 ± 0.014 | < 0.01 | 0.777 | 0.283 |
| **Prokaryotic (Bacteria)** |  |  |  |  |  |  |  |
| Acidobacteria | 0.013 ± 0.010 | 0.27 | 0.447 | 0.010 ± 0.006 | 0.07 | 0.157 | 0.418 |
| Actinobacteria | **0.023 ± 0.008** | **0.10** | **0.010** | **0.019 ± 0.008** | **0.16** | **0.047** | 0.277 |
| Alphaproteobacteria | 0.017 ± 0.009 | 0.13 | 0.185 | **0.021 ± 0.009** | **0.24** | **0.021** | 0.315 |
| Armatimonadetes | −0.012 ± 0.046 | 0.25 | 0.945 | 0.011 ± 0.033 | 0.13 | 0.898 | 0.335 |
| Bacteroidetes | 0.020 ± 0.030 | 0.24 | 0.297 | −0.011 ± 0.019 | 0.04 | 0.744 | 0.074 |
| Betaproteobacteria | 0.016 ± 0.010 | 0.19 | 0.154 | 0.005 ± 0.010 | 0.23 | 0.789 | 0.118 |
| Chlamydiae | 0.012 ± 0.046 | 0.09 | 0.966 | 0.021 ± 0.039 | 0.03 | 0.708 | 0.392 |
| Chloroflexi | 0.020 ± 0.027 | 0.14 | 0.650 | 0.041 ± 0.027 | 0.05 | 0.165 | 0.284 |
| Deltaproteobacteria | 0.030 ± 0.014 | 0.36 | 0.116 | 0.031 ± 0.018 | 0.16 | 0.074 | 0.477 |
| Firmicutes | 0.012 ± 0.015 | 0.07 | 0.198 | 0.029 ± 0.014 | 0.22 | 0.124 | 0.191 |
| Gammaproteobacteria | 0.040 ± 0.018 | 0.13 | 0.125 | 0.013 ± 0.019 | 0.16 | 0.502 | 0.094 |
| Gemmatimonadetes | **0.073 ± 0.024** | **0.25** | **0.018** | **0.043 ± 0.022** | **0.27** | **0.009** | 0.115 |
| Latescibacteria | −0.016 ± 0.077 | < 0.01 | 0.891 | 0.044 ± 0.067 | 0.12 | 0.662 | 0.271 |
| Nitrospirae | **0.056 ± 0.024** | **0.10** | **0.045** | 0.033 ± 0.022 | 0.20 | 0.118 | 0.168 |
| Parcubacteria | 0.099 ± 0.101 | 0.17 | 0.347 | 0.134 ± 0.094 | 0.19 | 0.215 | 0.370 |
| Planctomycetes | 0.017 ± 0.023 | 0.23 | 0.625 | 0.008 ± 0.015 | < 0.01 | 0.699 | 0.352 |
| Verrucomicrobia | 0.004 ± 0.016 | 0.28 | 0.957 | 0.008 ± 0.013 | 0.07 | 0.528 | 0.400 |
| **Fungi** |  |  |  |  |  |  |  |
| Ascomycota | **0.096 ± 0.016** | **0.33** | **< 0.001** | **0.092 ± 0.020** | **0.17** | **0.003** | 0.420 |
| Basidiomycota | **0.215 ± 0.044** | **0.32** | **< 0.001** | **0.280 ± 0.052** | **0.25** | **0.002** | 0.162 |
| Chytridiomycota | −0.067 ± 0.107 | 0.01 | 0.600 | 0.139 ± 0.152 | 0.24 | 0.205 | 0.114 |
| Glomeromycota | −0.023 ± 0.084 | 0.13 | 0.959 | −0.185 ± 0.187 | 0.19 | 0.161 | 0.142 |
| Zygomycota | 0.062 ± 0.057 | 0.05 | 0.422 | 0.103 ± 0.067 | 0.13 | 0.147 | 0.305 |
| **C degradation** |  |  |  |  |  |  |  |
| Starch | **0.027 ± 0.005** | **0.35** | **0.009** | **0.016 ± 0.004** | **0.18** | **0.036** | **0.032** |
| Hemicellulose | **0.018 ± 0.005** | **0.29** | **0.020** | **0.018 ± 0.004** | **0.18** | **0.031** | 0.493 |
| Pectin | **0.025 ± 0.005** | **0.35** | **0.005** | **0.021 ± 0.004** | **0.22** | **0.025** | 0.234 |
| Cellulose | **0.028 ± 0.006** | **0.24** | **0.011** | **0.024 ± 0.005** | **0.18** | **0.035** | 0.221 |
| Phospholipids | **0.060 ± 0.011** | **0.45** | **0.002** | **0.023 ± 0.007** | **0.14** | **0.039** | **< 0.001** |
| Terpenes | **0.017 ± 0.004** | **0.24** | **0.014** | 0.005 ± 0.004 | 0.02 | 0.391 | **0.004** |
| Chitin | **0.028 ± 0.006** | **0.31** | **0.016** | **0.023 ± 0.005** | **0.24** | **0.024** | 0.210 |
| Vanillin/Lignin | **0.029 ± 0.007** | **0.28** | **0.011** | **0.015 ± 0.006** | **0.14** | **0.043** | **0.023** |
| **Nutrient cycling genes** |  |  |  |  |  |  |  |
| N cycling | **0.024 ± 0.005** | **0.29** | **0.013** | **0.018 ± 0.005** | **0.18** | **0.029** | 0.127 |
| P utilization | **0.024 ± 0.006** | **0.32** | **0.012** | **0.019 ± 0.005** | **0.20** | **0.030** | 0.166 |
| S metabolism | **0.031 ± 0.006** | **0.35** | **0.008** | **0.020 ± 0.006** | **0.20** | **0.020** | **0.023** |

[1] The slopes were estimated using linear mixed-effects models (LMMs). *R*^2^ values represent the variances explained by the whole LMM model. The slopes are presented as a coefficient in fixed effect ± standard error in random effect.
[2] Statistical significance was determined using permutation tests with 1000 randomizations. Significant effects (*P <* 0.05) are indicated in bold.
[3] Statistical significance of the differences in TDR values between the increased precipitation treatment and the control was determined using permutation tests with 1000 randomizations.

**Table S8. Mantel test correlations between environmental variables and the structure of functional gene groups involved in soil carbon decomposition and nutrient (N, P, and S) cycling processes.**

| **Gene category**^[1]^ | **Substrate/Processes**^[2]^ | **Moisture** | **pH** | **NH_4_^+^** | **NO_3_^−^** | **TN** | **TOC** | **PR**^[3]^ | **ANPP** | **ER** | **GPP** | ***R*_a_** | ***R*_h_** |
| --- | --- | --- | --- | --- | --- | --- | --- | --- | --- | --- | --- | --- | --- |
| C degradation | Starch | **0.41***** | **0.38***** | < 0.01 | −0.08 | −0.07 | −0.06 | **0.13^#^** | **0.13^#^** | 0.14 | **0.20^#^** | 0.02 | **0.20*** |
|  | Hemicellulose | **0.39***** | **0.40***** | < 0.01 | −0.08 | −0.07 | −0.07 | **0.15^#^** | **0.12^#^** | 0.11 | **0.17^#^** | < 0.01 | **0.18*** |
|  | Pectin | **0.32***** | **0.45***** | −0.01 | −0.06 | −0.08 | −0.09 | **0.22^#^** | 0.08 | 0.11 | **0.18^#^** | −0.02 | **0.15^#^** |
|  | Cellulose | **0.38***** | **0.37***** | −0.03 | −0.07 | −0.08 | −0.08 | **0.12^#^** | **0.14^#^** | 0.13 | **0.18*** | > −0.01 | **0.15*** |
|  | Phospholipids | **0.28***** | **0.39***** | −0.02 | −0.06 | −0.05 | −0.06 | **0.23^#^** | 0.04 | 0.06 | 0.12 | −0.03 | **0.19*** |
|  | Terpenes | **0.39***** | **0.34***** | 0.01 | −0.07 | −0.05 | −0.04 | **0.11^#^** | **0.11^#^** | 0.11 | **0.16*** | 0.05 | **0.24***** |
|  | Chitin | **0.37***** | **0.42***** | −0.01 | −0.07 | −0.10 | −0.09 | **0.18^#^** | **0.11^#^** | 0.13 | **0.18^#^** | −0.02 | **0.17*** |
|  | Vanillin/Lignin | **0.38***** | **0.37***** | −0.03 | −0.08 | −0.10 | −0.10 | **0.13^#^** | **0.17^#^** | 0.23 | **0.31*** | 0.02 | **0.23**** |
| N cycling | Ammonification | **0.35***** | **0.38***** | 0.04 | −0.01 | −0.10 | −0.09 | **0.12^#^** | 0.08 | 0.08 | **0.15^#^** | 0.01 | **0.12^#^** |
|  | Anammox | **0.10^#^** | **0.19*** | −0.01 | 0.01 | −0.09 | −0.07 | > −0.01 | 0.04 | −0.08 | −0.10 | −0.06 | 0.04 |
|  | Assimilatory N reduction | **0.41***** | **0.36***** | < 0.01 | −0.08 | −0.08 | −0.08 | **0.14^#^** | **0.10^#^** | 0.16 | **0.21^#^** | −0.02 | **0.12^#^** |
|  | Denitrification | **0.38***** | **0.44***** | −0.01 | −0.06 | −0.09 | −0.09 | **0.19^#^** | **0.11^#^** | 0.11 | **0.17^#^** | −0.01 | **0.19*** |
|  | Dissimilatory N reduction | **0.36***** | **0.34***** | −0.02 | −0.05 | −0.08 | −0.07 | **0.09^#^** | **0.13^#^** | 0.18 | **0.27*** | 0.01 | **0.18*** |
|  | Nitrification | **0.29***** | **0.23**** | 0.12 | −0.02 | −0.07 | −0.05 | **0.09^#^** | −0.01 | −0.06 | −0.05 | −0.06 | **0.12^#^** |
|  | N_2_ fixation | **0.40***** | **0.34***** | 0.03 | −0.06 | −0.12 | −0.11 | **0.14^#^** | 0.09 | −0.01 | 0.04 | −0.03 | **0.21***** |
| P utilization | Phosphorus oxidation | **0.21**** | **0.27***** | 0.02 | −0.05 | −0.10 | −0.10 | 0.07 | 0.02 | 0.03 | 0.05 | −0.07 | 0.06 |
|  | Phytic acid hydrolysis | **0.42***** | **0.30***** | 0.05 | −0.11 | −0.06 | −0.04 | **0.10^#^** | 0.08 | 0.18 | **0.23*** | 0.05 | **0.19*** |
|  | Polyphosphate degradation | **0.44***** | **0.40***** | 0.01 | −0.07 | −0.07 | −0.06 | **0.13^#^** | **0.12^#^** | 0.12 | **0.19*** | 0.03 | **0.20*** |
|  | Polyphosphate synthesis | **0.44***** | **0.36***** | 0.01 | −0.06 | −0.07 | −0.07 | **0.14^#^** | **0.14^#^** | 0.11 | **0.19*** | 0.02 | **0.24***** |
| S metabolism | Sulfite Reduction | **0.46***** | **0.40***** | > −0.01 | −0.07 | −0.08 | −0.07 | **0.16^#^** | **0.11^#^** | 0.15 | **0.22*** | 0.03 | **0.24*** |
|  | Sulfide Oxidation | **0.15*** | **0.39***** | −0.07 | 0.08 | −0.06 | −0.07 | **0.17^#^** | 0.08 | 0.02 | 0.09 | −0.06 | 0.04 |
|  | S Oxidation | **0.40***** | **0.43***** | −0.01 | −0.07 | −0.07 | −0.08 | **0.15^#^** | **0.11^#^** | 0.10 | **0.15^#^** | −0.01 | **0.14^#^** |

[1] Functional gene group structures were determined by GeoChip hybridization.
[2] Values represent Mantel test *r* statistics, with significance represented by *** *P <* 0.001, ** *P <* 0.01, * *P <* 0.05, ^#^ *P <* 0.10. Significant correlations (*P <* 0.05) are highlighted in bold. All *p*-values were adjusted using the false discovery rate (FDR) method.
[3] PR: plant richness. ANPP: above-ground net primary production (estimated as above-ground plant biomass). ER: ecosystem respiration. GPP: gross primary productivity. *R*_a_: autotrophic respiration. *R*_h_: heterotrophic respiration.

**Table S9. Mantel test correlations between environmental variables and the structure of significantly (*P <* 0.05) increased functional genes in Fig. 3a.**

| **Gene category**^[1]^ | **Gene**^[2]^ | **Moisture** | **pH** | **NH_4_^+^** | **NO_3_^−^** | **TN** | **TOC** | **PR**^[3]^ | **ANPP** | **ER** | **GPP** | ***R*_a_** | ***R*_h_** |
| --- | --- | --- | --- | --- | --- | --- | --- | --- | --- | --- | --- | --- | --- |
| C degradation | *amyA* | **0.40***** | **0.38***** | < 0.01 | −0.08 | −0.07 | −0.06 | **0.13^#^** | **0.13^#^** | 0.14 | **0.20*** | 0.02 | **0.20*** |
|  | *cda* | **0.36***** | **0.35***** | −0.02 | −0.03 | −0.07 | −0.07 | **0.12^#^** | **0.12^#^** | 0.13 | **0.17^#^** | −0.02 | **0.17*** |
|  | *glucoamylase* | **0.45***** | **0.32***** | 0.04 | −0.08 | −0.06 | −0.05 | **0.10^#^** | **0.13^#^** | 0.17 | **0.21*** | 0.03 | **0.19*** |
|  | *nplT* | **0.34***** | **0.41***** | < 0.01 | −0.04 | −0.07 | −0.07 | **0.19^#^** | 0.07 | < 0.01 | 0.06 | −0.03 | **0.22*** |
|  | *ara* | **0.33***** | **0.38***** | −0.03 | −0.09 | −0.11 | −0.11 | **0.13^#^** | **0.11^#^** | 0.10 | **0.15^#^** | −0.04 | **0.12^#^** |
|  | *xyla* | **0.37***** | **0.38***** | −0.03 | −0.05 | −0.07 | −0.07 | **0.16^#^** | **0.14^#^** | 0.15 | **0.23*** | −0.02 | **0.19*** |
|  | *xylanase* | **0.44***** | **0.39***** | 0.04 | −0.09 | −0.04 | −0.03 | **0.13^#^** | **0.15^#^** | 0.11 | **0.17^#^** | 0.06 | **0.24***** |
|  | *pectate_lyase* | **0.30**** | **0.34***** | 0.04 | −0.07 | −0.05 | −0.07 | **0.26^#^** | < 0.01 | −0.02 | 0.07 | 0.03 | **0.26*** |
|  | *pectinase* | **0.26**** | **0.31**** | 0.01 | −0.11 | −0.09 | −0.05 | **0.19^#^** | **0.15^#^** | 0.24 | **0.34**** | 0.03 | **0.14*** |
|  | *Pg* | **0.45***** | **0.30***** | 0.03 | 0.04 | −0.05 | −0.05 | **0.10^#^** | 0.07 | −0.03 | 0.04 | 0.09 | **0.26***** |
|  | *pme* | **0.33***** | **0.42***** | 0.03 | −0.11 | −0.09 | −0.09 | **0.19^#^** | 0.04 | 0.07 | 0.10 | 0.02 | **0.16*** |
|  | *Pme* | **0.40***** | **0.42***** | 0.02 | −0.11 | −0.05 | −0.05 | **0.20^#^** | **0.13^#^** | < 0.01 | 0.07 | 0.01 | **0.22*** |
|  | *rgl* | **0.34***** | **0.39***** | < 0.01 | −0.06 | −0.09 | −0.09 | **0.16^#^** | **0.09^#^** | 0.13 | **0.18^#^** | > −0.01 | **0.10^#^** |
|  | *axe* | **0.46***** | **0.25***** | −0.01 | −0.04 | −0.04 | −0.03 | **0.10^#^** | **0.14*** | 0.09 | **0.15*** | 0.05 | **0.19*** |
|  | *endoglucanase* | **0.36***** | **0.37***** | −0.02 | −0.05 | −0.07 | −0.07 | **0.13^#^** | **0.12^#^** | 0.10 | **0.15^#^** | 0.03 | **0.15*** |
|  | *exoglucanase* | **0.34***** | **0.24***** | −0.03 | −0.11 | −0.06 | −0.04 | 0.09 | **0.20*** | 0.16 | **0.21*** | −0.01 | **0.16*** |
|  | *phospholipase_C* | **0.36***** | **0.32***** | −0.05 | −0.08 | −0.08 | −0.08 | **0.13^#^** | **0.11^#^** | 0.01 | 0.08 | −0.04 | **0.17*** |
|  | *phospholipase_D* | **0.21*** | **0.37***** | −0.02 | −0.04 | −0.04 | −0.05 | **0.22^#^** | 0.02 | 0.05 | 0.09 | −0.04 | **0.15*** |
|  | *lmo* | **0.32***** | **0.12^#^** | 0.10 | −0.04 | 0.09 | 0.10 | 0.01 | **0.10^#^** | < 0.01 | 0.06 | 0.12 | **0.28***** |
|  | *acetylglucosaminidase* | **0.38***** | **0.37***** | < 0.01 | −0.08 | −0.10 | −0.10 | **0.15^#^** | **0.12^#^** | 0.13^#^ | **0.16^#^** | −0.01 | **0.17*** |
|  | *chitin_deacetylase* | **0.31***** | **0.20**** | 0.03 | −0.08 | −0.02 | < 0.01 | 0.02 | **0.17*** | **0.24*** | **0.25**** | 0.04 | **0.13*** |
|  | *chitinase* | **0.36***** | **0.43***** | −0.02 | −0.07 | −0.10 | −0.09 | **0.19^#^** | 0.10 | 0.13 | **0.19^#^** | −0.03 | **0.17*** |
|  | *mnp* | **0.36***** | **0.35***** | −0.03 | −0.02 | −0.08 | −0.08 | **0.14^#^** | **0.12^#^** | 0.13 | **0.18*** | −0.02 | **0.21*** |
|  | *vanA* | **0.43***** | **0.40***** | −0.01 | −0.12 | −0.09 | −0.08 | **0.14^#^** | **0.11^#^** | 0.17 | **0.23*** | 0.05 | **0.25**** |
| C fixation | *aclb* | **0.34***** | **0.35***** | 0.02 | 0.01 | −0.03 | −0.02 | **0.12^#^** | **0.17*** | 0.08 | **0.13^#^** | 0.07 | **0.10^#^** |
|  | *codh* | **0.24**** | **0.31**** | 0.07 | −0.06 | −0.02 | −0.04 | **0.12^#^** | 0.04 | 0.07 | 0.12 | −0.01 | 0.09 |
|  | *pcc* | **0.39***** | **0.38**** | −0.02 | −0.10 | −0.08 | −0.06 | **0.11^#^** | **0.15^#^** | 0.22 | **0.30*** | 0.01 | **0.20*** |
| N cycling | *gdh* | **0.14*** | **0.23*** | −0.06 | 0.11 | −0.08 | −0.08 | 0.04 | 0.04 | 0.03 | 0.07 | −0.03 | −0.01 |
|  | *nirA* | **0.44***** | **0.21**** | 0.05 | −0.13 | −0.07 | −0.05 | **0.14^#^** | **0.10^#^** | **0.21^#^** | **0.27**** | −0.02 | **0.12*** |
|  | *narG* | **0.37***** | **0.42***** | −0.01 | −0.06 | −0.08 | −0.08 | **0.18^#^** | **0.13^#^** | 0.12 | **0.19^#^** | −0.02 | **0.18*** |
|  | *nirK* | **0.40***** | **0.40***** | 0.01 | −0.09 | −0.09 | −0.09 | **0.17^#^** | **0.10^#^** | 0.07 | **0.13^#^** | 0.03 | **0.20*** |
|  | *nirS* | **0.32***** | **0.44***** | −0.02 | −0.03 | −0.09 | −0.09 | **0.19^#^** | 0.07 | 0.06 | 0.12 | −0.03 | **0.19*** |
|  | *norB* | **0.44***** | **0.40***** | 0.05 | −0.07 | −0.06 | −0.05 | **0.21^#^** | **0.10^#^** | 0.03 | 0.10 | 0.02 | **0.26**** |
|  | *nosZ* | **0.36***** | **0.45***** | −0.01 | −0.05 | −0.09 | −0.09 | **0.19^#^** | **0.11^#^** | 0.13 | **0.19^#^** | −0.02 | **0.15*** |
|  | *napA* | **0.38***** | **0.30***** | −0.03 | −0.05 | −0.06 | −0.06 | **0.12^#^** | **0.13^#^** | 0.17 | **0.25**** | < 0.01 | **0.18*** |
| P utilization | *phytase* | **0.42***** | **0.30***** | 0.05 | −0.11 | −0.06 | −0.04 | **0.10^#^** | 0.08 | 0.18 | **0.23*** | 0.05 | **0.19*** |
|  | *ppk* | **0.44***** | **0.36***** | 0.01 | −0.06 | −0.07 | −0.07 | **0.14^#^** | **0.14^#^** | 0.11 | **0.19*** | 0.02 | **0.24**** |
|  | *ppk2* | **0.47***** | **0.35***** | 0.04 | −0.07 | −0.05 | −0.04 | **0.11^#^** | **0.12^#^** | 0.11 | **0.18*** | 0.04 | **0.24**** |
| S metabolism | *cysJ* | **0.39***** | **0.39***** | −0.03 | −0.06 | −0.08 | −0.08 | **0.17^#^** | **0.10^#^** | 0.17 | **0.23*** | 0.01 | **0.18*** |
|  | *dsrB* | **0.51***** | **0.41***** | 0.01 | −0.07 | −0.08 | −0.06 | **0.11^#^** | **0.12^#^** | 0.12 | **0.19*** | 0.06 | **0.26**** |
|  | *sir* | **0.42***** | **0.39***** | −0.03 | −0.04 | −0.07 | −0.05 | 0.08 | 0.09 | 0.22 | **0.32**** | 0.09 | **0.25***** |
|  | *soxA* | **0.32***** | **0.40***** | −0.03 | −0.04 | −0.02 | −0.03 | **0.15^#^** | **0.09^#^** | 0.15 | **0.25*** | 0.04 | **0.19*** |
|  | *soxB* | **0.35***** | **0.29***** | −0.03 | 0.07 | −0.02 | −0.03 | 0.02 | 0.05 | 0.03 | 0.07 | 0.05 | **0.11*** |
|  | *soxY* | **0.40***** | **0.42***** | < 0.01 | −0.07 | −0.07 | −0.07 | **0.16^#^** | **0.10^#^** | 0.09 | **0.14^#^** | −0.01 | **0.15*** |

[1] Significantly (*P <* 0.05) increased functional gene structures were determined by GeoChip hybridization.
[2] Values represent Mantel test *r* statistics, with significance represented by *** *P <* 0.001, ** *P <* 0.01, * *P <* 0.05, ^#^ *P <* 0.10. Significant correlations (*P <* 0.05) are highlighted in bold. All *p*-values were adjusted using the false discovery rate (FDR) method.
[3] PR: plant richness. ANPP: above-ground net primary production (estimated as above-ground plant biomass). ER: ecosystem respiration. GPP: gross primary productivity. *R*_a_: autotrophic respiration. *R*_h_: heterotrophic respiration.

**Table S10. Significance of environmental variables in explaining microbial community taxonomic and functional variations in the CCA.**

| **Environmental Variables**^[1]^ | **Functional genes (GeoChip)** | | **Prokaryotes (16S rRNA)** | | **Fungi (ITS)** | |
| --- | --- | --- | --- | --- | --- | --- |
|  | ***F*** | ***P***^[2]^ | ***F*** | ***P*** | ***F*** | ***P*** |
| Time | **15.38** | **0.001** | **1.544** | **0.001** | **2.019** | **0.001** |
| Moisture | **5.033** | **0.001** | 1.175 | 0.073 | **1.262** | **0.001** |
| pH | **4.156** | **0.001** | **1.375** | **0.004** | **1.327** | **0.001** |
| Temperature | 1.472 | 0.125 | 1.072 | 0.159 | 0.884 | 0.786 |
| NO_3_^−^ | **2.409** | **0.015** | **1.177** | **0.041** | 1.049 | 0.314 |
| NH_4_^+^ | **1.899** | **0.033** | 1.124 | 0.100 | 1.025 | 0.362 |
| PR | 1.029 | 0.374 | 1.054 | 0.174 | **1.288** | **0.001** |
| ANPP | 1.791 | 0.053 | 1.080 | 0.155 | **1.209** | **0.013** |
| ER | 1.517 | 0.091 | 1.050 | 0.226 | **1.152** | **0.041** |
| GPP | 0.932 | 0.478 | 1.040 | 0.275 | 1.070 | 0.178 |
| Overall model | **3.562** | **0.001** | **1.169** | **0.014** | **1.228** | **0.001** |

[1] PR: plant richness. ANPP: above-ground net primary production (estimated as above-ground plant biomass). ER: ecosystem respiration. GPP: gross primary productivity. *R*_a_: autotrophic respiration. *R*_h_: heterotrophic respiration.
[2] Significant variables (*P <* 0.05) are given in bold.
